# The Early Apoptotic DNA Fragmentation Targets a Small Number of Specific Open Chromatin Regions

**DOI:** 10.1371/journal.pone.0005010

**Published:** 2009-04-06

**Authors:** Miriam Di Filippo, Giorgio Bernardi

**Affiliations:** Laboratory of Molecular Evolution, Stazione Zoologica Anton Dohrn, Naples, Italy; Texas A&M University, United States of America

## Abstract

We report here that early apoptotic DNA fragmentation, as obtained by using an entirely new approach, is the result of an attack at a small number of specific open chromatin regions of interphase nuclei. This was demonstrated as follows: (i) chicken liver was excised and kept in sterile tubes for 1 to 3 hours at 37°C; (ii) this induced apoptosis (possibly because of oxygen deprivation), as shown by the electrophoretic nucleosomal ladder produced by DNA preparations; (iii) low molecular-weight DNA fragments (∼200 bp) were cloned, sequenced, and shown to derive predominantly from genes and surrounding 100 kb regions; (iv) a few hundred cuts were produced, very often involving the same chromosomal sites; (v) at comparable DNA degradation levels, micrococcal nuclease (MNase) also showed a general preference for genes and surrounding regions, but MNase cuts were located at sites that were quite distinct from, and less specific than, those cut by apoptosis. In conclusion, the approach presented here, which is the mildest and least intrusive approach, identifies a preferred accessibility landscape in interphase chromatin.

## Introduction

Chromatin structure plays a fundamental role in the regulation of gene expression (see [1], [2] for reviews). A classical approach to investigate chromatin structure since the work of Hewish and Burgoyne [3] is to study the regions that are accessible to DNA-degrading enzymes. This approach was widely used on interphase nuclei whose isolation, however, potentially affects chromatin structure. Here we investigated the targeting of cuts early in DNA fragmentation by apoptosis of chicken liver cells and discovered that a small number of specific open chromatin regions were attacked with strong preference.

Apoptosis, or programmed cell death, is a highly ordered mechanism of cell elimination which is involved in many processes such as embryogenesis, metamorphosis and tissue homeostasis [4], 5. At the molecular level, a degradation of proteins and DNA is observed [6], [7]. The latter involves several endogenous DNases (see [8], [9] for reviews), that break DNA into high molecular weight (HMW) fragments ranging from 50 to 300 kb (as analyzed by pulse-field electrophoresis [10]). These molecules are subsequently further degraded to low molecular weight (LMW) oligonucleosome-sized fragments. It is generally accepted that the endonuclease activity(ies) responsible for the generation of the large fragments is different from that catalyzing internucleosomal DNA fragmentation [11] and that the two stages of HMW and LMW DNA fragmentation are discrete processes that can be separated experimentally (see [12] for a review). The cleavage in the internucleosomal regions generates DNA fragments having sizes corresponding to multiples of 180–200 bp, giving rise to a typical “ladder” (when analyzed by conventional gel electrophoresis [6], [11]), similar to that obtained after micrococcal nuclease (MNase) digestion [13]. While both DNA degradations preferentially cut genes and their surroundings, most remarkably apoptotic cuts are quite distinct from, and more specific than, MNase cuts. This indicates a very different accessibility of open chromatin in apoptotic cells and isolated nuclei.

## Results

### Apoptotic DNA ladder formation

**Figure 1:**
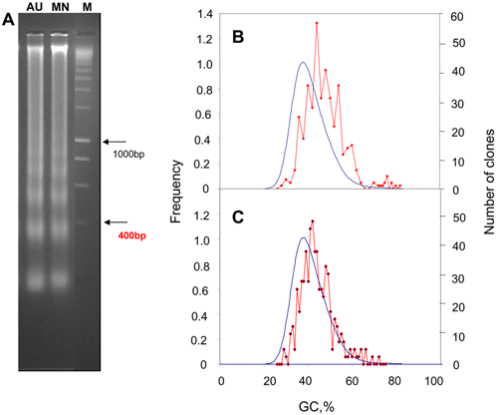
Nucleosome ladder and compositional distribution of low molecular weight (LMW) DNA fragments. (A) Chicken liver was cut into small pieces and immediately put in sterile tubes for 1 hr at 37°C (AU). Chicken liver nuclei were digested with 300 U of MNase for 30 sec. at 37°C (MN). DNA was purified from both preparations and run on 2% agarose gel producing a “nucleosome ladder”. The marker Smart Ladder Eurogentec is also shown (M). Compositional distribution of chicken DNA and of low molecular weight (LMW) DNA fragments after autolysis (B) or MNase digestion (C). The GC level of the LMW DNA fragments (average length ∼200 bp, red line) was superimposed on the GC profile of the chicken genome (blue line), as assessed at a window size of 200 bp. The frequency of the segments (×10^−6^) was indicated on the left and right ordinates for the chicken genome and the LMW DNA fragments respectively.

Apoptosis was induced by keeping excised chicken liver in sterile tubes at 37°C. DNA degradation was investigated just after the first appearance of the nucleosomal ladder when the vast majority of DNA was still present as a very high molecular weight band and some DNA had not even entered the gel (Figure 1A). A similar pattern of DNA degradation was produced when isolated chicken liver nuclei were briefly digested with micrococcal nuclease (MNase; Figure 1A). Expectedly, no degradation was observed before the addition of the enzyme, as already observed in previous work from other laboratories.

### GC level of cloned fragments

In order to obtain further information on the apoptotic LMW DNA fragments, we cloned and sequenced 370 fragments having a size of ∼200 bp. The average GC level of the cloned fragments, 46.8%, was higher than the average GC level of the chicken genome, 41%, but not quite as high as the average GC level of the small DNA fragments as directly extracted from the gel (49.6% GC). This result was in all likelihood due to a cloning bias against the GC-richest DNA fragments. When the distribution of the cloned fragments in 1% GC bins was superimposed on the GC profile of total chicken genome obtained using a window size of 256 base pairs (Figure 1B), the clones were shown to be enriched in GC-rich sequences. In fact, the clones covered a range of 30% to 75% GC, indicating that a small number of very GC-rich fragments could still be cloned in spite of the bias mentioned above. A similar result, with a slightly less pronounced shift, was found with 245 cloned MNase fragments in the 200 bp range (Figure 1C).

### Localization of the clones in chicken isochores

We localized all the sequences cloned from both apoptosis and MNase degradation on chicken chromosomes. This localization obviously could not be done for the clones (representing about 20% of all clones) that corresponded to unassembled scaffolds, unsequenced microchromosomes and repeats. This reduced the number of localized clones to 317 and 193 for apoptotic and micrococcal degradation, respectively. Taking into consideration that a number of clones from each degradation coincided in chromosome location, the numbers of “non-redundant” clones were 200 and 165 for the two degradations. The coordinates of these clones on chromosomes are presented in Table S1. The full maps of both series of cuts on chicken chromosomes are displayed on 24 panels (Figure S1) along with the GC profiles of chicken chromosomes and the corresponding isochore maps.

**Figure 2:**
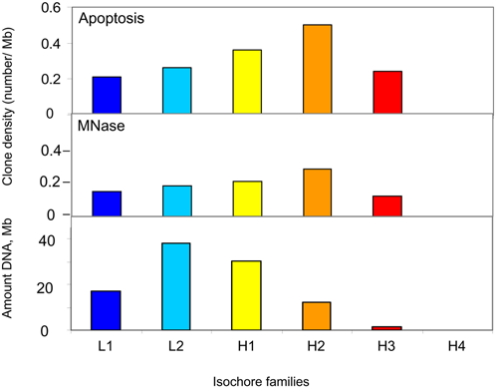
Density of apoptotic and MNase LMW cloned DNA fragments in isochore families. The bottom panel shows the amount of DNA in each isochore family of the chicken genome.

The clone density in the isochore families as defined by Costantini et al [14] was found to show a similar trend in both degradations, in that it increased with increasing GC level, to decrease in the very GC-rich family H3 (Figure 2A and 2B). The latter result is, in all likelihood, due to the loss of some DNA fragments from the isochore family H3, because of the cloning bias against GC-rich fragments and of the unavailability of sequences from some microchromosomes (and also from macrochromosome W). As for the first reason, it should be pointed out that, in spite of the cloning bias, clones higher than 60% GC were obtained from both degradations, as already mentioned (see Figure 1B and 1C). As far as the second reason is concerned, it should be stressed that microchromosomes are GC-richer than macrochromosomes [15] and that cloned fragments have a higher density in the former compared to the latter (see Figure S2).

### Identification of the sequences

**Figure 3:**
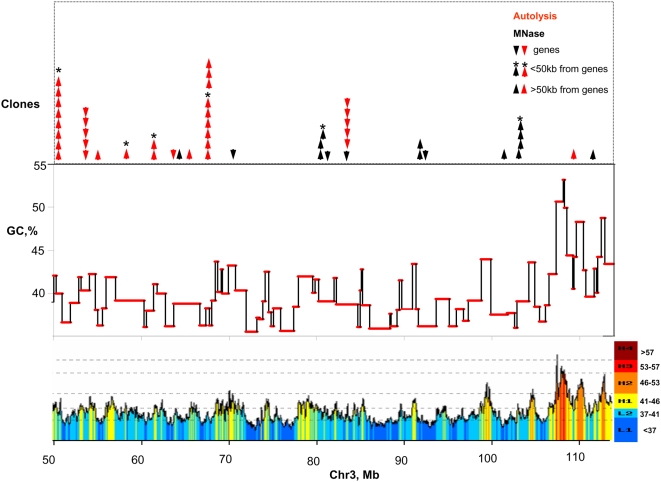
Mapping of apoptotic and MNase cuts on chicken chromosome 1. The GC level and the gene density in the isochores are shown for the telomeric 60 Mb of the p arm of chicken chromosome 3. The arrows indicate the cuts (in red for the autolysis and in black for MNase digestion). The down- and up- pointed arrows ↓ ↑ indicate cuts in genes or in sequences within 50 kb (asterisks) or 100 kb from genes. Cut mapping on all other chromosomes is reported in Figure S1.

**Figure 4:**
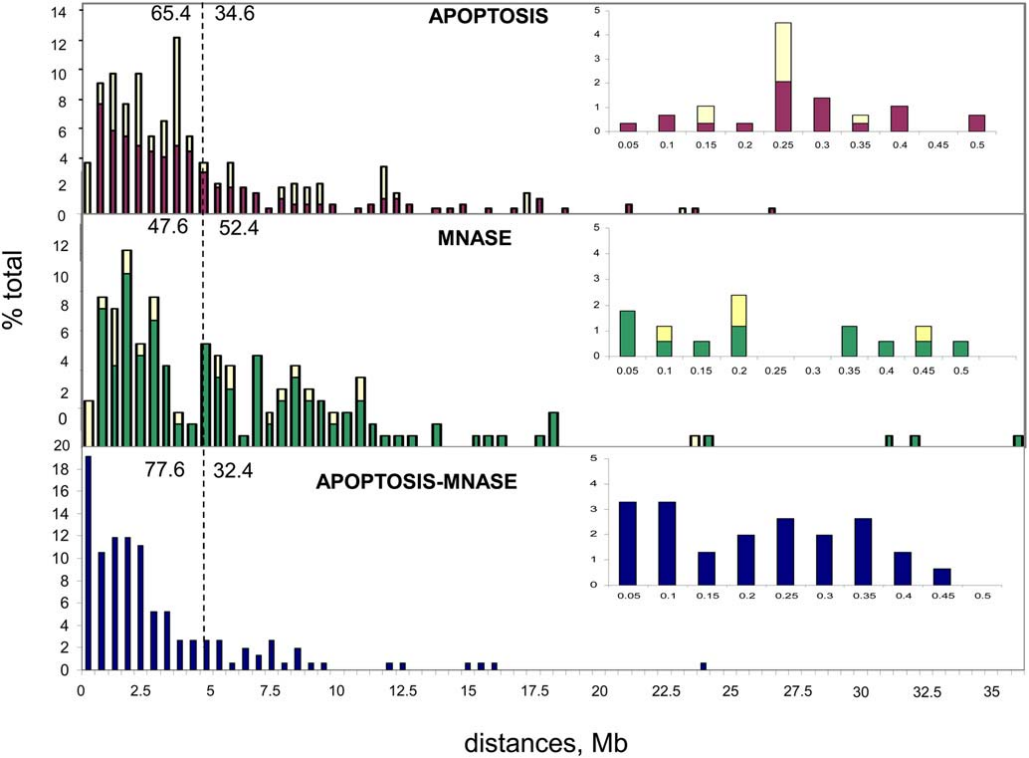
Map distances (as % of total distances) among clones from autolysis and MNase. Histogram are presented in Mb bins (top yellow segments refer to redundant clones; inserts refer to distances lower than 500 kb).

The maps of apoptotic and MNase cuts provided several interesting results: (i) In both degradations a preference was observed for genes, as well as narrow (<50 kb) and wide (50–100 kb) chromosomal surroundings of genes; this preference concerned about 60% of all cleavage sites (see Table 1); another 26% or 19% of cuts (in apoptotic and MNase degradation, respectively) were located at intergenic sequences farther away from genes. (ii) In both cases, a preference was also found for interspersed repeats mainly from the CR1 families; while these repeats obviously could not be mapped, they are known to be preferentially located in GC-rich genome regions [15], [16]. (iii) “Redundant” clones, namely clones mapping at identical chromosomal sites, were present in both cases, but they were more than twice as abundant when derived from apoptosis than from MNase (see Figures 3 and 4 and Table 2); incidentally, since “redundant” clones from repeated sequences could not be mapped, overall redundancies were underestimated. (iv) In the case of microchrosomes several of them (14, 19, 21, 25) were only cut by apoptosis, other ones (15, 22, 24, 26, 27) only by MNase (see Figure S2). (v) Minimal distances between cuts and their nearest neighbors ranged from 0.65 kb to 26,000 kb (with an average of 4,600 kb) in the case of apoptosis and from 1.80 kb to 36,000 kb (with an average of 5,700 kb) in that of MNase; in the first case, distances lower than 4 Mb were found for 65.4% of all fragments, *vs* only 47.6% in the second (see Figure 4). Interestingly minimal distances among autolytic cuts and MNase cuts ranged from 26 kb to 23,800 kb (with an average of 2,900 kb; and 77.6% of distances below 4 Mb; see Figure 4 and Table 2). The distributions of distances in the 0–500 kb range are also shown in Figure 4, the short 50–100 kb range being more represented in the distances among different cuts (6.5%) than in the distances within the “same” cuts (1.7% for apoptotic or 2.8% MNase cuts).

**Table 1:** Sequences cleaved by autolysis and MNase.

	APOPTOSIS	MNASE
	%	%
**GENES+SURROUNDINGS**	**60.3**	**61.6**
Genes	39.2	32.6
<50 kb from genes	16.5	18
>50 kb <100 kb from genes	4.6	11
**INTERGENIC (>100 kb FROM GENES)**	**25.7**	**18.8**
**REPEATS**	**14.3**	**19.5**
CR1	8.6	4.9
GGXHOI family satellite	1.1	8.2
GGL ERV LTR-repeats	0.5	2.5
Mariner1b_GG family	1.3	1.6
other repeats	2.8	2.3

**Table 2:** Number of clones, redundancies, sites, and minimal distances between the location of the clones derived from apoptotic or MNase degradation and between the two kinds of cuts.

	Autolysis	MNase	Autolysis/Mnase
**Number of Clones**	317	193	
**Site redundant clones**	117 **(36.9%)**	28 **(14.5%)**	
**Sites**	200	165	
**Distances, Mb**	4.6	5.7	2.9
<4 Mb	1.8	1.5	1.5
**Relative amounts (%)**			
**<4 Mb**	65.4	47.6	77.6
**<0.01 Mb**	1.7	2.8	6.5

**Figure 5:**
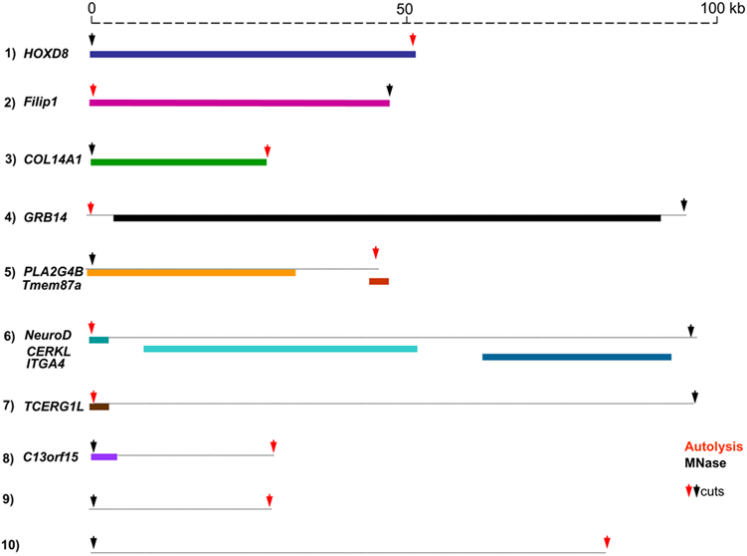
Scheme of autolytic and MNase cuts within 100 kb. Maps of MNase and autolytic cuts (black and red arrows, respectively) and of genes (colored stretches) located at (and between) cuts. See Table S3 online for the origin and description of the genes presented in the Figure.

An interesting situation was the one in which the two cuts were within 100 kb (see Figure 4 and Table 2). A shown in Figure 5, out of ten such cases, four (1 to 4) were characterized by cuts in the same genes (in one of them, cuts were just outside the gene sequence which is known to lack the 5′ end); two (5 and 6) had one cut in one gene and another cut in another gene (again in number 6 one cut was just outside the end of the gene), two (7 and 8) had a cut in one gene only, and two (9 and 10) had two cuts outside genes. In all likelihood, the two cuts concerned the same domain (or loop) of chromatin, being less than 50 Kb (in six cases) and less than 100 Kb (in four cases) apart.

### Sequence specificity of apoptotic endonucleases and MNase

The preferential 5′ and 3′ terminal dinucleotides of the clones obtained from apoptotic degradation were CT/AG, GG/CC and TG/CA; Figure S3A), without any preference for purines or pyrimidines (Figure S3C).

In the case of MNase digestion a sequence specificity was found for TG/CA dinucleotides, namely for purine/pyrimidine dinucleotides (Figure S3B and S3D). The analysis of the next nucleotides also indicated a specificity for the palindromic trinucleotides TGC/GCA (see Table S2). These results confirm and extend the previous findings that MNase cleaves DNA preferentially in (A–T)n regions (n≥1) flanked by a 5′C or G [17]–[21]. Interestingly, in both apoptotic and MNase degradation the doublets TG and CA were the most represented in terminal positions. In the apoptotic case other dinucleotides were, however, more abundant than in the MNase degradation. Since the larger distances could be, in the case of MNase, due to the fact that the number of non-redundant clones was lower (165 vs 200; see Table 2), 165 randomly chosen apoptotic clones were investigated and distances were found to be comparable to those of MNase clones (5.4 Mb).

## Discussion

### Apoptosis via autolysis

Apoptosis is usually induced in cell cultures by chemical or physical agents, such as X-rays, UV-light, bleomycin, ectoposide, dimethylsulphoxide (DMSO). In our work, apoptosis was simply induced by leaving tissues in sterile tubes, an entirely new way of inducing apoptosis. In this case, the most likely trigger that activates programmed cell death is deprivation of oxygen. Indeed, the time after which the nucleosomal ladder is already visible (30′) is too short for other causes (e.g. lack of nutrients or of growth factors) to be responsible for it. Since this induction of apoptosis is perfectly reproducible, we propose it as an alternative approach to induction by physical or chemical agents.

Our approach has at least two implications. The first one is that oxigen deprivation most likely kills cells by apoptosis, a point of obvious interest in medicine. The second implication derives from the preference for the open chromatin around active genes and of the double-break nature of apoptotic cuts [22] . Indeed, this implies that the cell death sentence in all likelihood takes place at the very beginning of apoptosis, when the very first 50–300 kb fragments are produced (see Introduction).

### Chromatin structure and its accessibility to nucleases

It is known from previous work that the GC-rich, gene-rich isochores are characterized by a more expanded (open) configuration than the GC-poor, gene-poor isochores in the interphase nucleus of human and chicken [23]–[25]. The present investigations go beyond this initial observation in that they provide information on the structure of open chromatin, as investigated in cells undergoing apoptosis and in isolated nuclei. When the open chromatin regions were explored at a nucleotide resolution, a number of features appeared to be shared by the two degradations. Indeed, both of them were shown to target the GC-rich, gene-rich regions of the genome, as well as repeats that are preferentially located in those regions (see Table 1). Incidentally, such preference is also true for DNase I hypersensitive sites as assessed in human isochores [26].

The minimal distances between sites from each degradation were found to be very large. On a first instance, this result can be understood on the account that in both cases an early phase of degradation was explored. Undoubtedly, additional sites could have been discovered if losses of GC-rich fragments had not been incurred because of cloning problems. However, the abundance of redundant clones, namely of clones having an identical chromosomal location, in both apoptotic and MNase degradation (but especially in the former one), indicates that the most preferred sites were clearly detected in both cases.

Distances between cuts by the two degradations were found to be shorter than those within each set of sites, indicating a trend for a very broad “clustering”, without however, a single coincidence of the two kinds of cuts. Distances generally remained, however, very large, 90% of the cuts being separated by more than 1 Mb on the average (see Figure 4). The distribution of sites in different isochore families was very unequal, cuts being exceedingly rare in GC-poor isochores, which are known to be in a very compact chromatin structure. In this case apparently the large 50–300 kb fragments are only rarely produced and are unavailable for the further degradation to oligonucleosomes.. The patterns of cuts caused by the two degradations are different. Actually, a majority (55%) of the two kinds of cuts concern different isochores. Moreover, there are remarkable differences in the microchromosomes attacked by the two degradations (Figure S2). Since this is most unlikely due to the different specificities of the two enzymes, because these specificities are very low and largely similar, the most plausible explanation is that chromatin structures from tissues undergoing apoptosis and from isolated nuclei expose different open regions as sites available for enzymatic attack. Differences may be due to the procedure used in the preparation of nuclei, which includes the use of Ca^2+^, Mg^2+^, spermine and spermidine (two agents that strongly interact with DNA), as opposed to the much milder apoptotic degradation. In other words, what we suggest is that the most accessible chromatin regions woul be artefactually modified by the cations and the basic organic compounds, so presenting different regions of the open chromatin to micrococcal nuclease. On the other hand, in the latter case one cannot rule out the action of proteolysis which might also concern chromatin [27], [28]. However, if this unlikely event would have occurred under our experimental conditions this would have led to a broadening of the accessible regions and would indicate that, in the absence of such hypothetical proteolytic attack, the accessible regions would be even narrower than those found. The possibility that an endogenous nuclease is already located close to some (the most redundant) cleavage sites cannot be ruled out, but is unlikely. While these points deserve further work, clearly apoptotic degradation opens up a new area of exploration of the most accessible open chromatin structures in the interphase nucleus.

In any case the total number of sites, even taking into account those missed because of cloning problems, is in the range of a few hundreds, those of specifically targeted sites (as indicated by redundant clones) being at the lower end of the range. It is quite conceivable that the most accessible open chromatin regions which are repeatedly targeted by apoptotic degradation are different in different cell types, as suggested by preliminary experiments pointing to different rates of degradations in different tissues. In other words, the approach developed in this work should provide an overall view of the most accessible sites in interphase nuclei from different cell types.

## Methods

### Autolysis and DNA extraction

Samples of tissues and blood were taken from adult chickens and immediately stored in sterile tubes for different times and at different temperatures. Autolysis was stopped by extracting DNA using the Genomix DNA extraction Kit (Talent). After different time lapses (30 min to 24 hours), at different temperatures (4°C, room temperature and 37°C), DNA was extracted, purified and analyzed by electrophoresis on 2% agarose gels, thus generating a nucleosome ladder.

In the case of liver autolysis at 4°C, DNA digestion was extremely slow and there was no evidence of a nucleosome ladder even after 24 hours. At room temperature, the ladder started to form after 10 hours and was distinctly evident after 24 hours. At 37°C, the nucleosome ladder began to appear after 1 hour and DNA was hydrolyzed to oligonucleotides after 8 hours. Preliminary results indicated that under the standard condition of 37°C, 1 hr, liver, kidney and lung were faster digested than brain, muscle, heart and pancreas. Erythrocytes were resistant to degradation, even after 24 hours of autolysis at 37°C. In this case a striking resistance to MNase was also evident.

### Nuclei preparation and MNase digestion

Nuclei from the chicken tissues were prepared by a modified Hewish and Burgoyne [3] method as described by Kornberg et al [29]. Nuclei were then digested with MNase, 300 U/ml for 30 sec to 10 min at 37°C, the first time lapse being chosen as the standard procedure. Digestion was stopped by adding 0.5 M EDTA. DNA was extracted as mentioned above.

### Nucleosome ladder resolution by agarose gel electrophoresis

The samples were run in 2% agarose gels and visualized by staining with ethidium bromide.

### High-performance liquid chromatography (HPLC) analysis

LMW DNA fragments obtained by autolysis or MNase digestion were eluted from the gels using QIAquick Gel Extraction Kit and analyzed by HPLC as described by Varriale et al [30].

### Cloning and sequencing LMW DNA fragments

DNA fragments, as released by either autolysis or MNase digestion, were filled-in by the Klenow fragment enzyme and ligated in PCR-II vector using Topo Cloning blunt-ends Kit (Invitrogen). The recombinant clones were detected by blue/white screening, and sequenced using T7 and M13 reverse universal primers in Applied Biosystems 37300 DNA analyzer.

### Data analysis

The average GC level of the sequences was calculated by a computer program. The UCSC Genome Bionformatics Site contains the reference sequence and working draft assemblies for a large collection of genomes. In the case of the chicken genome, the May 2006 *Gallus gallus* draft assembly produced by the Genome Sequencing Center at the Washington University School of Medicine in St. Louis, MO, USA (WUSTL) was used. The DNA fragments cloned and their chromosome coordinates were identified by blast searching using URL: http://www.genome.ucsc.edu. The density of clones in the isochore families was calculated, on the basis of their locations and the coordinates of chicken isochores [14], by dividing the number of clones in each isochore family by the size (% of the genome size) of the family. The gene density in isochore families of a set of 12,663 chicken genes (retrieved from Ensembl) and the GC profiles of chicken chromosomes, are those reported by Costantini et al [14].

## Supporting Information

Figure S1 Clone density on chicken chromosomes. The chromosome location for all clones, for (A) autolysis and MNase digestion (B) respectively, was obtained by blast searching, using the URL: http://www.genome.ucsc.edu. The clone density was calculated as frequency of clones for chromosome / length of chromosome. A higher density of clones was found on microchromosomes.
(7.86 MB PDF)

Figure S2 Mapping of apoptotic and MNase cuts on chicken chromosomes. The cut location, gene density and the GC level in the isochores (as in the Figure 3) are shown on all chicken choromosomes sequenced.
(0.14 MB PDF)

Figure S3 Terminal dinucleotides analysis. Frequencies of the terminal dinucleotides of the autolytic sequences cloned after autolysis (A) and MNase digestion (C). The contribution of the clones (blue bars) and of redundant clones (white bars) in the dinucleotides were plotted together for each dinucleotide. All pairs of terminal dinucleotides after autolysis (B) and MNase digestion (D) are classified in the three possible groups: purine/purine (pur/pur), pyrimidine/pyrimidine (pyr/pyr), and purine/pyrimidine or viceversa (pyr/pur).
(0.69 MB TIF)

Table S1 (0.36 MB DOC)

Table S2 (0.04 MB DOC)

Table S3 (0.04 MB DOC)
