# Supplementary material for: The Early Apoptotic DNA Fragmentation Targets a Small Number of Specific Open Chromatin Regions
Source: PLoS One. 2009 Apr 6;4(4):e5010. doi: 10.1371/journal.pone.0005010 (PMC2661134; doi:10.1371/journal.pone.0005010)

Figure S1

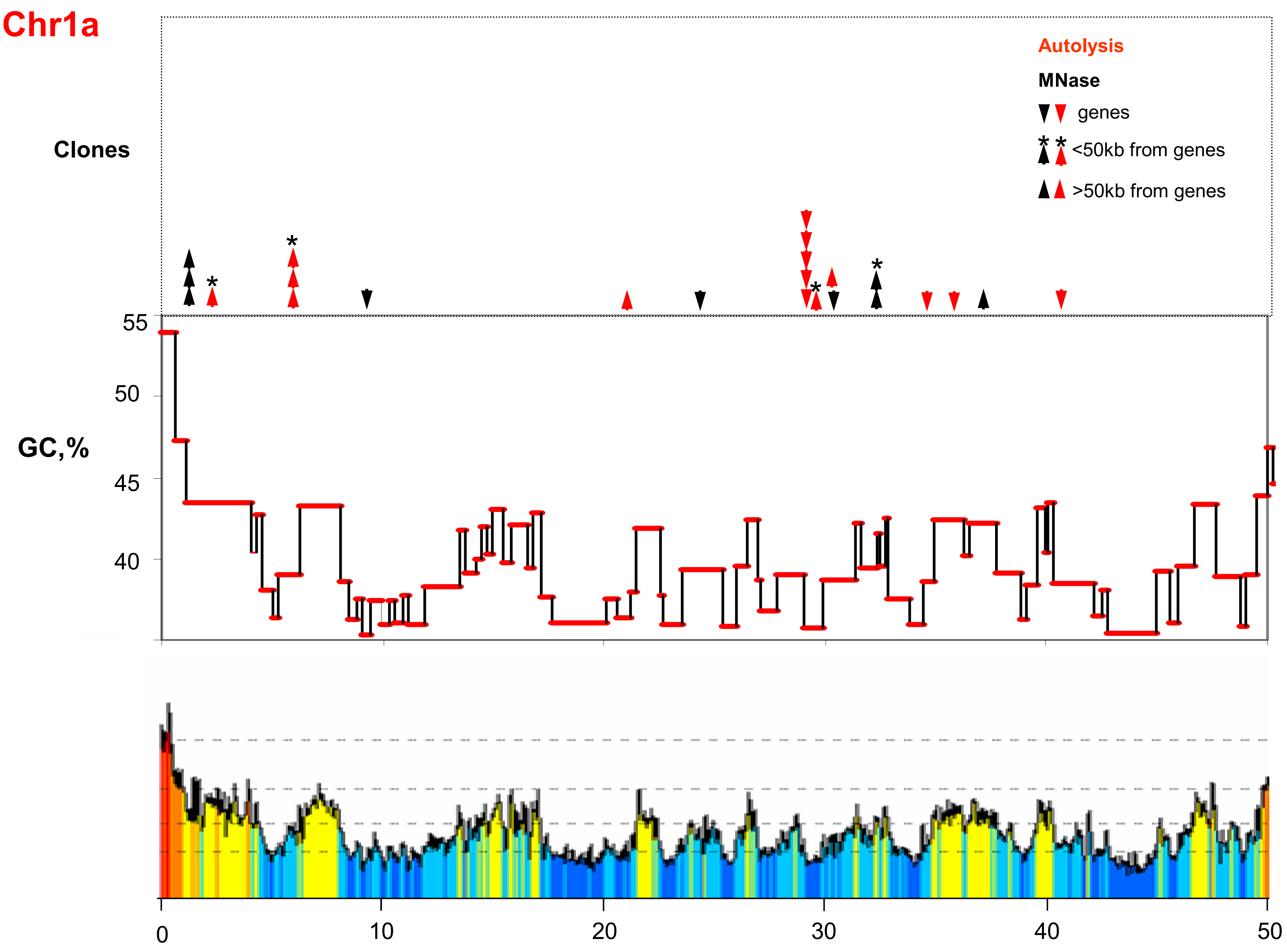

Chr1a
Autolysis
MNase
genes
<50kb from genes
>50kb from genes
Clones
GC,%
55
50
45
40
0
10
20
30
40
50

Chr1b

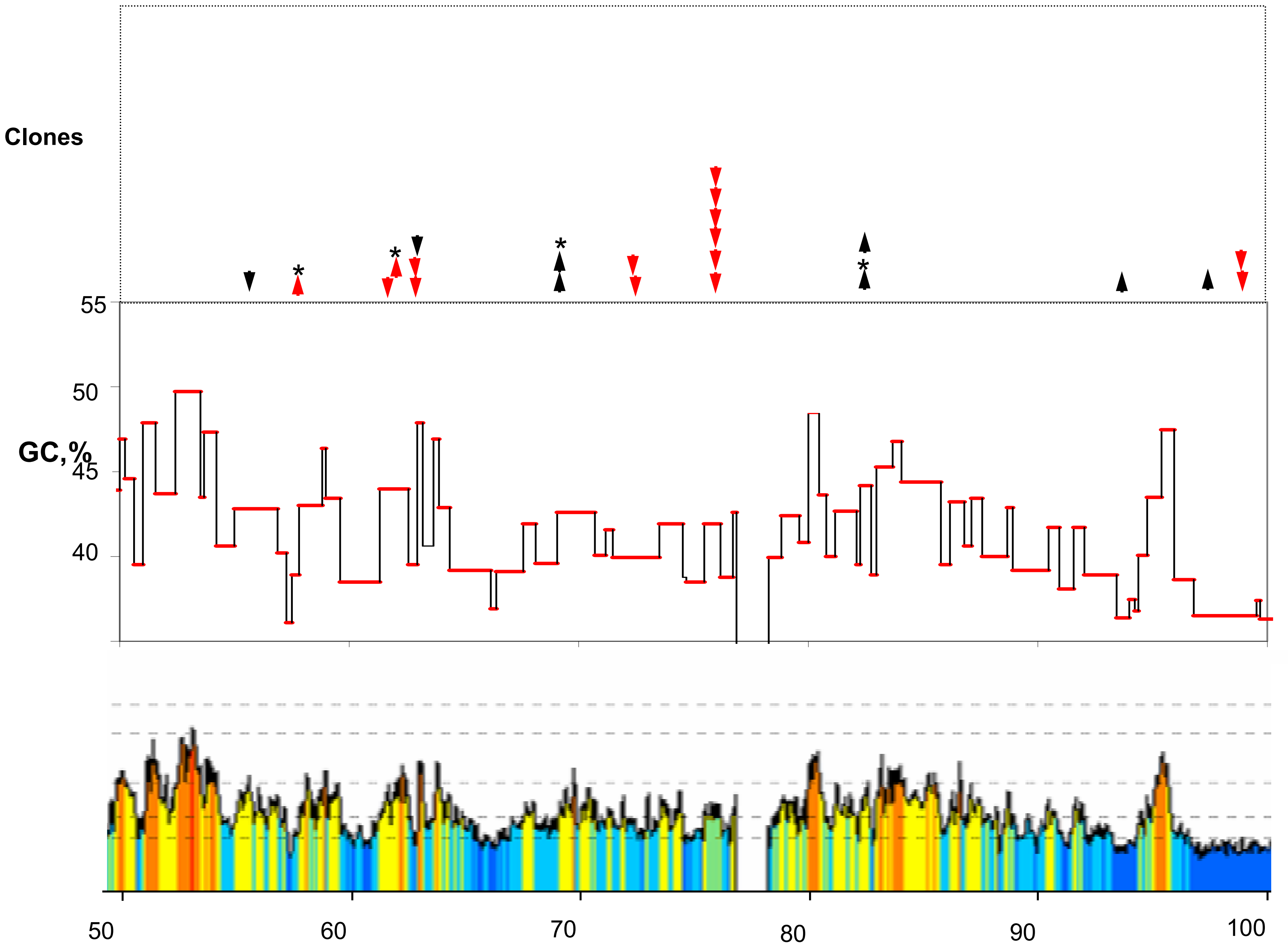

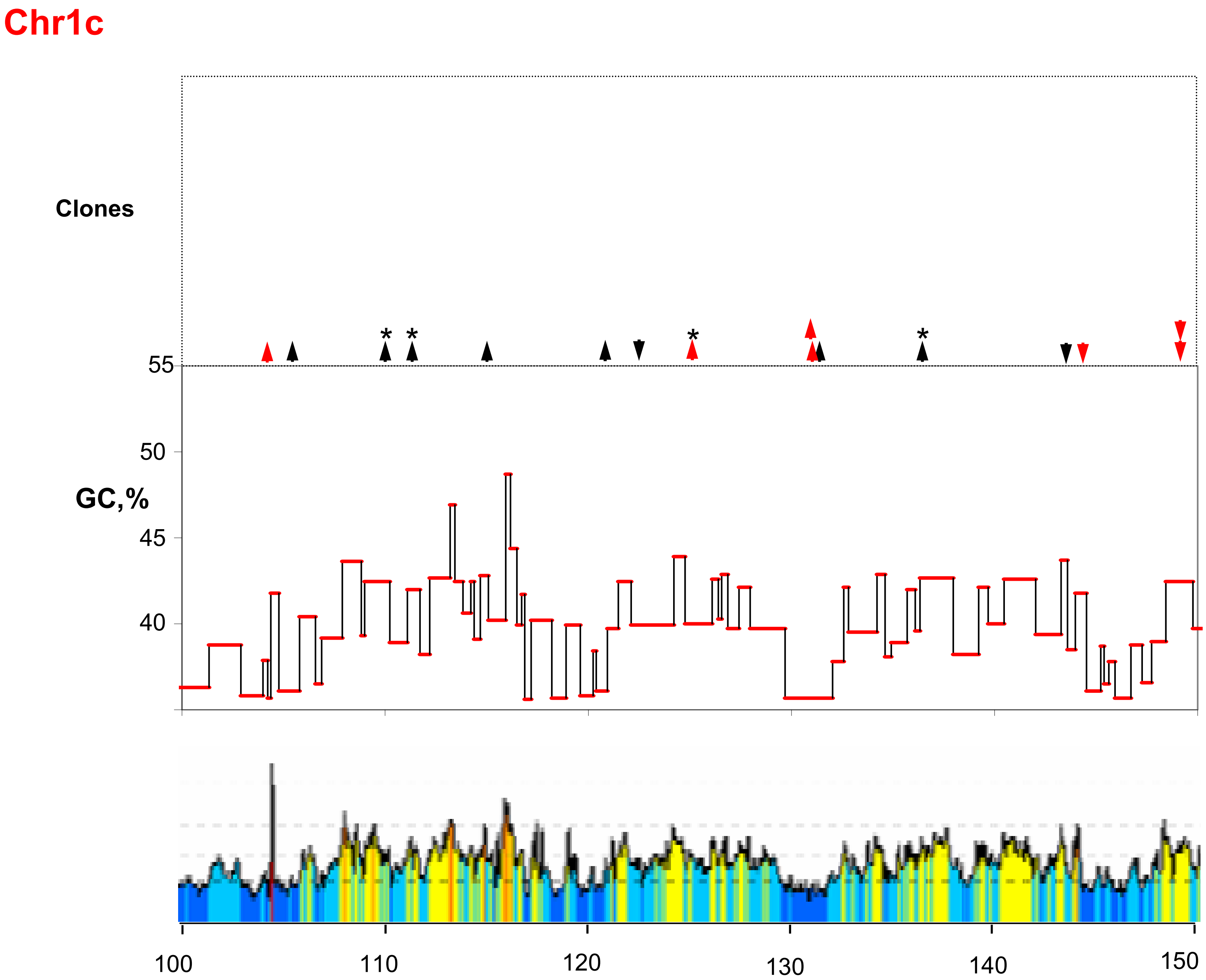

Chr1c
Clones
*
*
*
*
55
50
GC,%
45
40
100
110
120
130
140
150

Chr1d

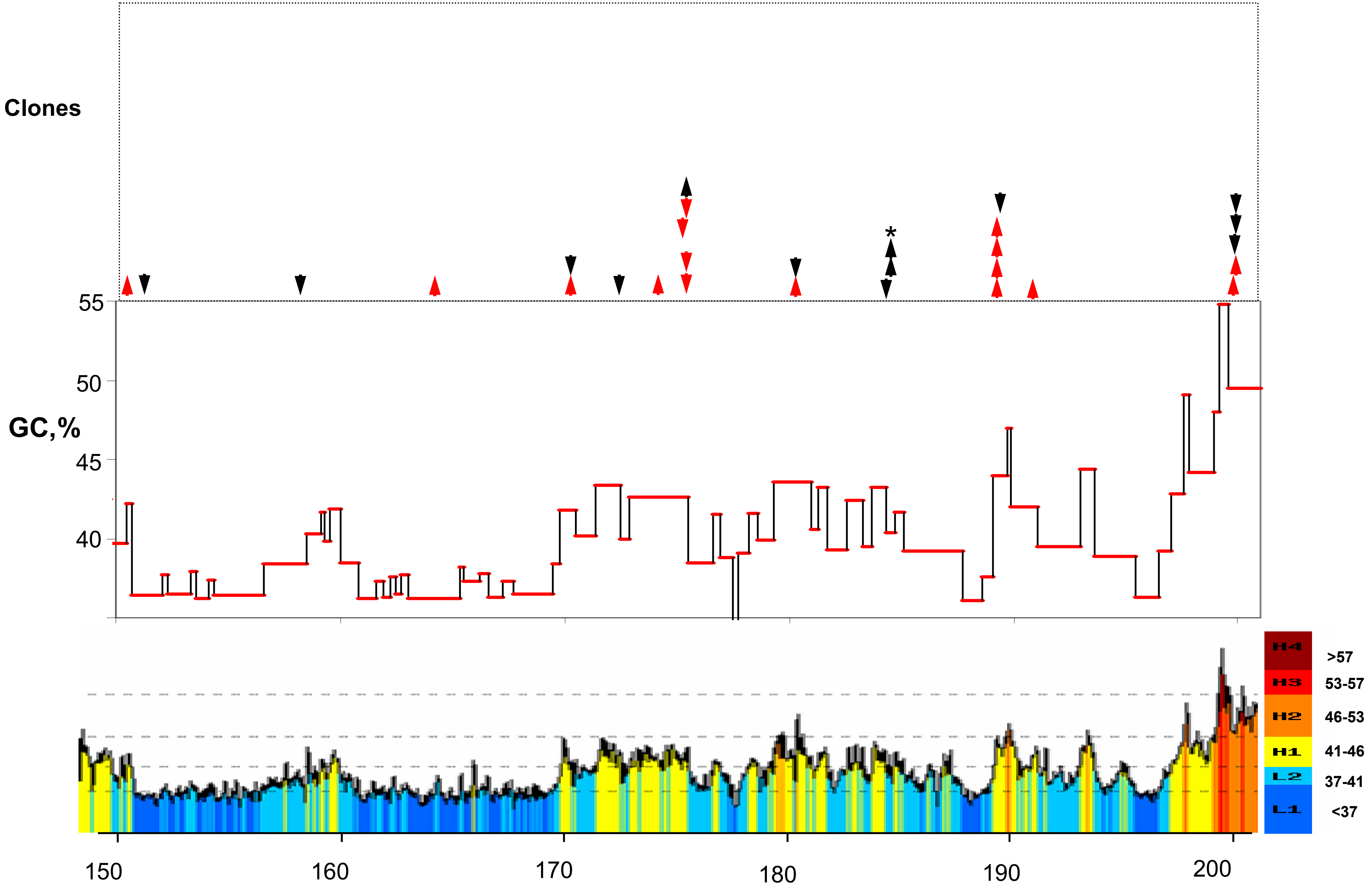

Clones
*
55
50
GC,%
45
40
H4
>57
H3
53-57
H2
46-53
H1
41-46
L2
37-41
L1
<37
150
160
170
180
190
200

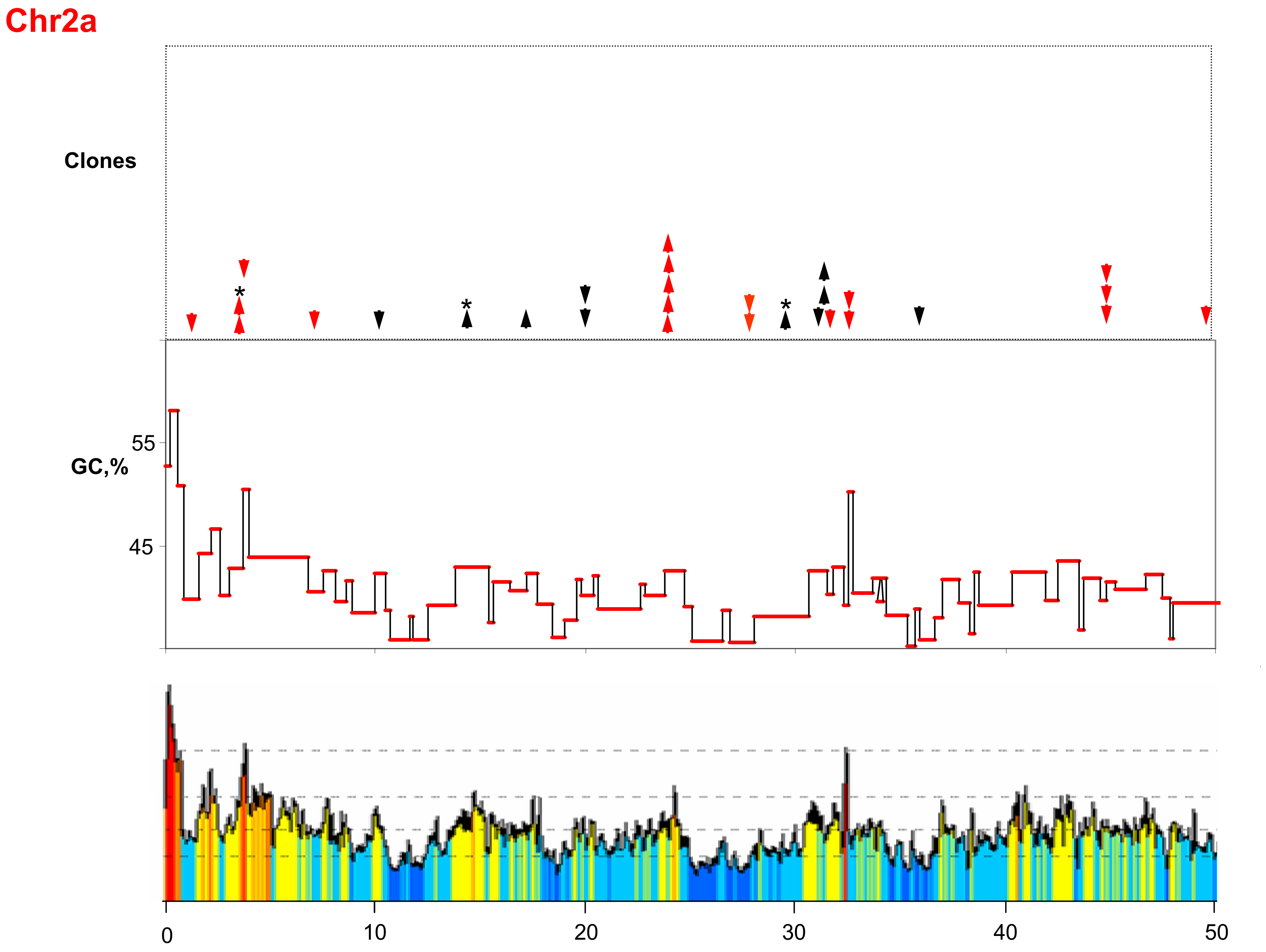

Chr2a
Clones
GC,%
55
45
0
10
20
30
40
50

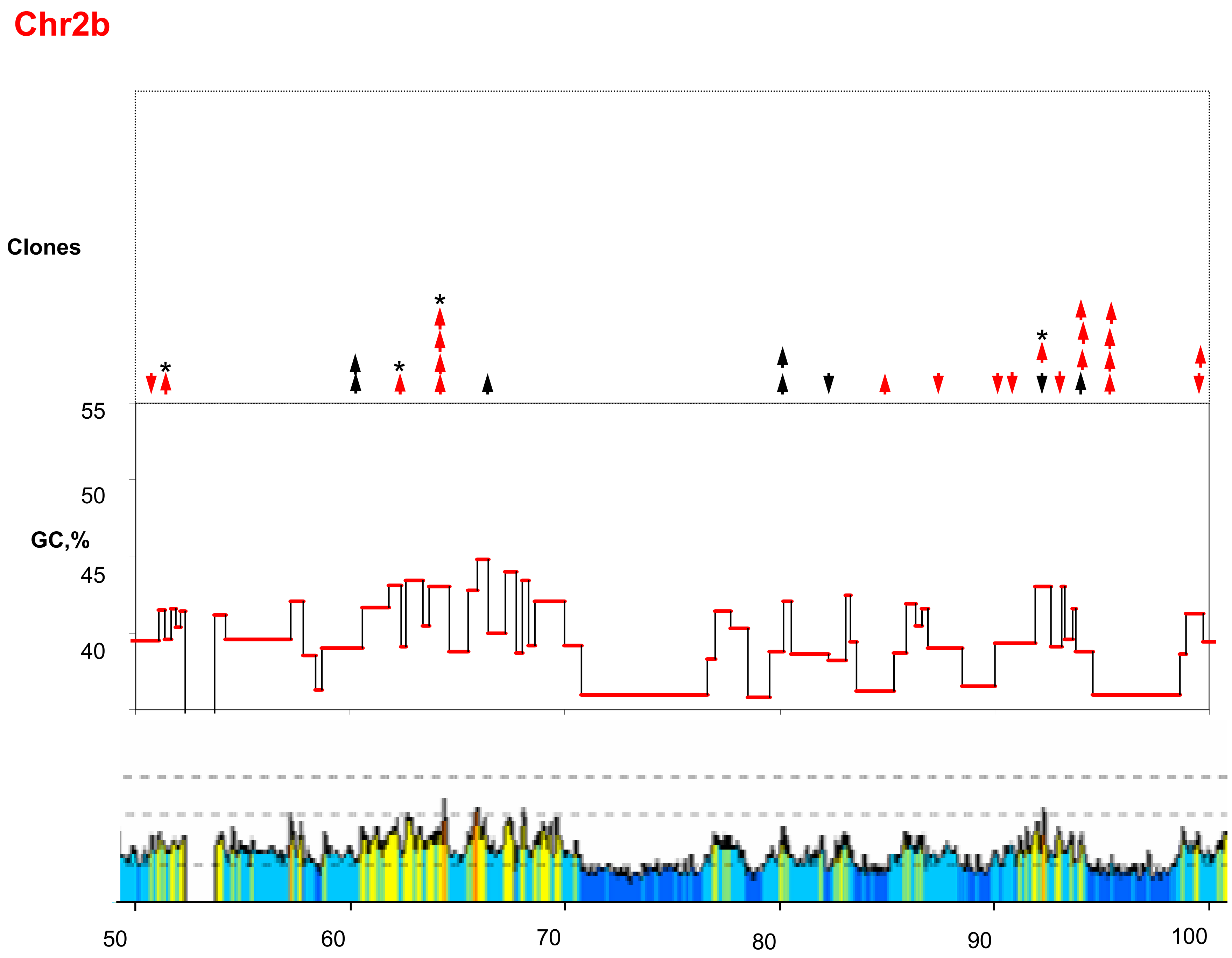
Chr2b
Clones
GC,%
55
50
45
40
50
60
70
80
90
100

Chr2c

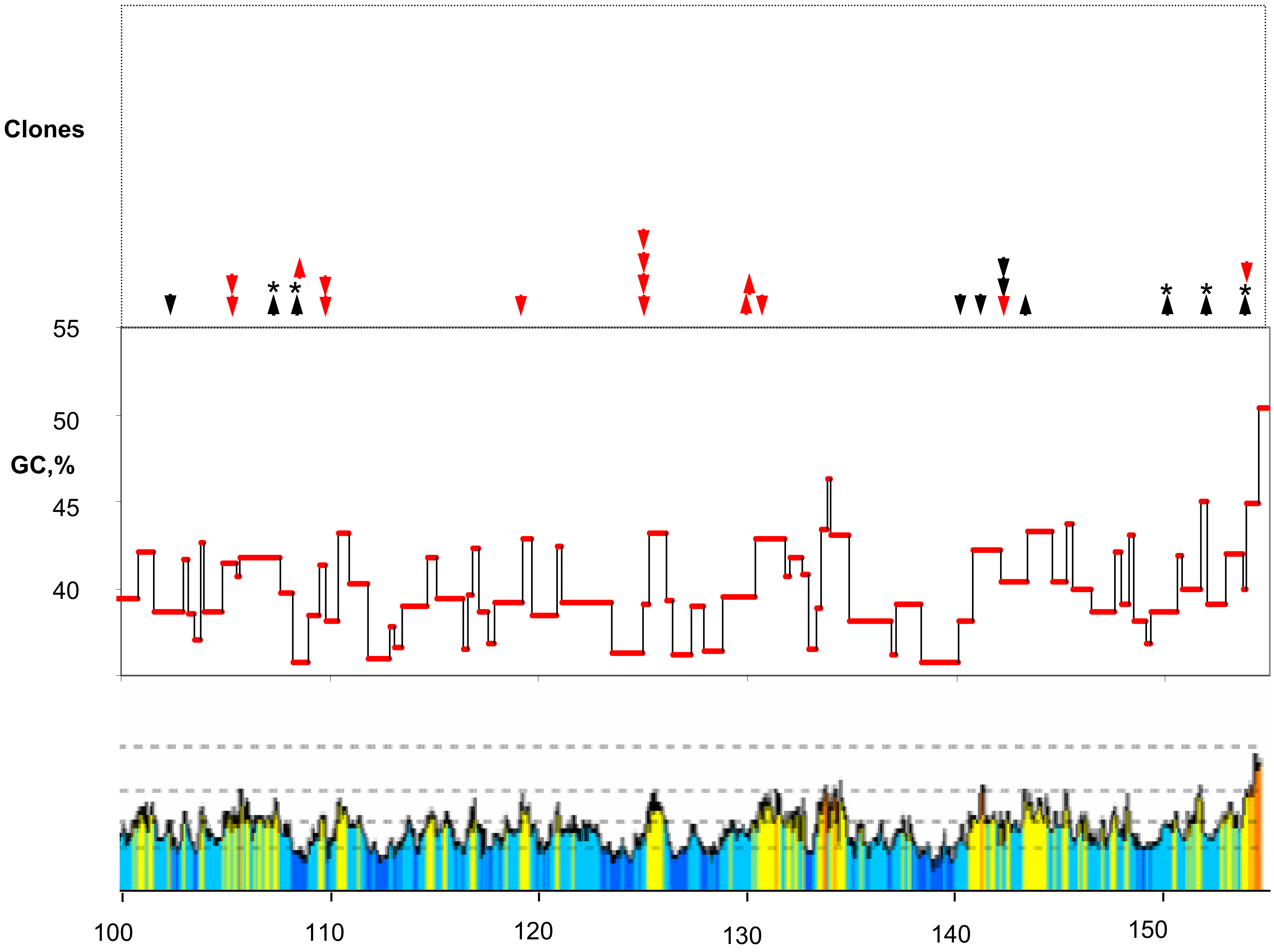

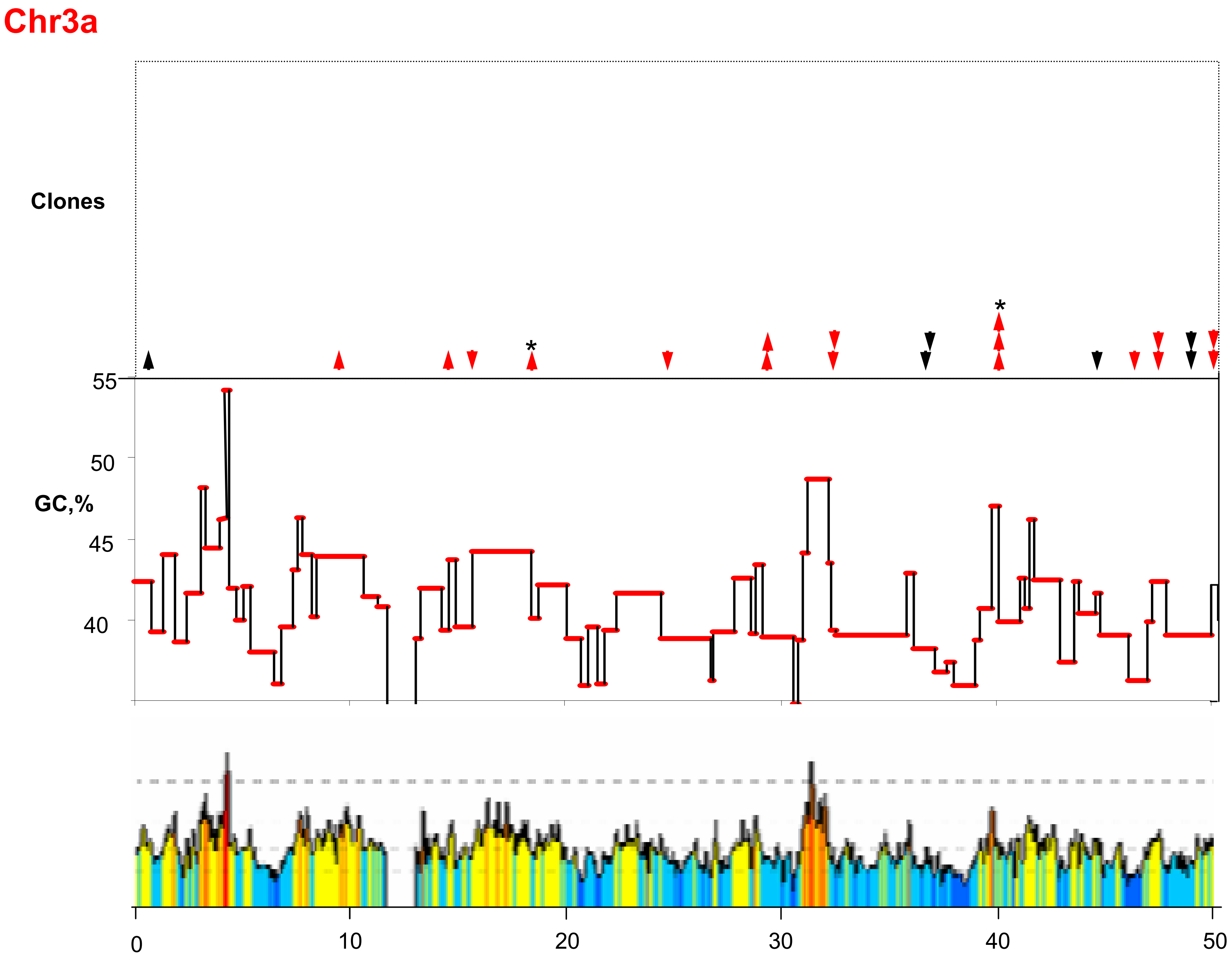

Chr3a
Clones
GC,%
55
50
45
40
*
*
0
10
20
30
40
50

Chr3b

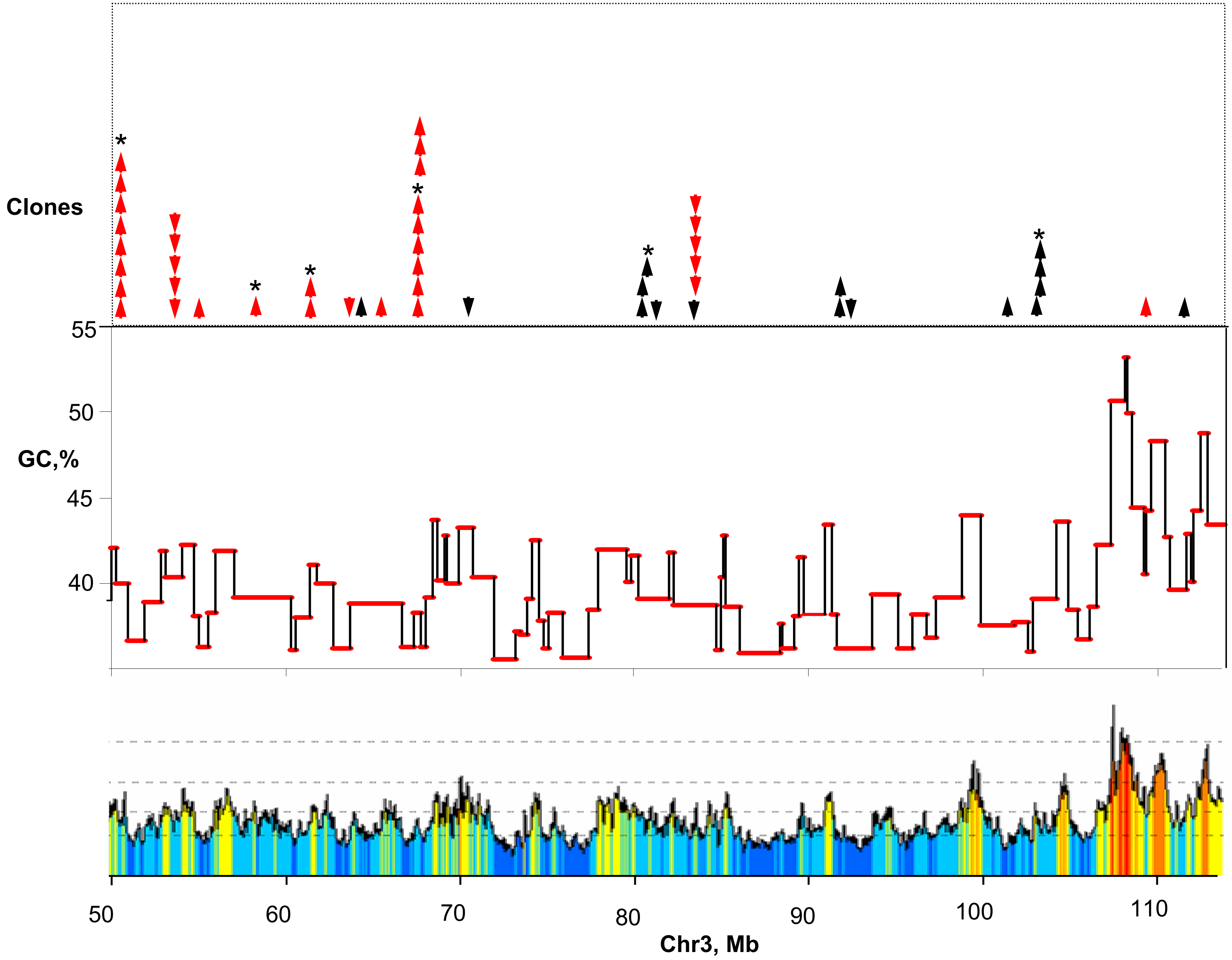

Chr4a

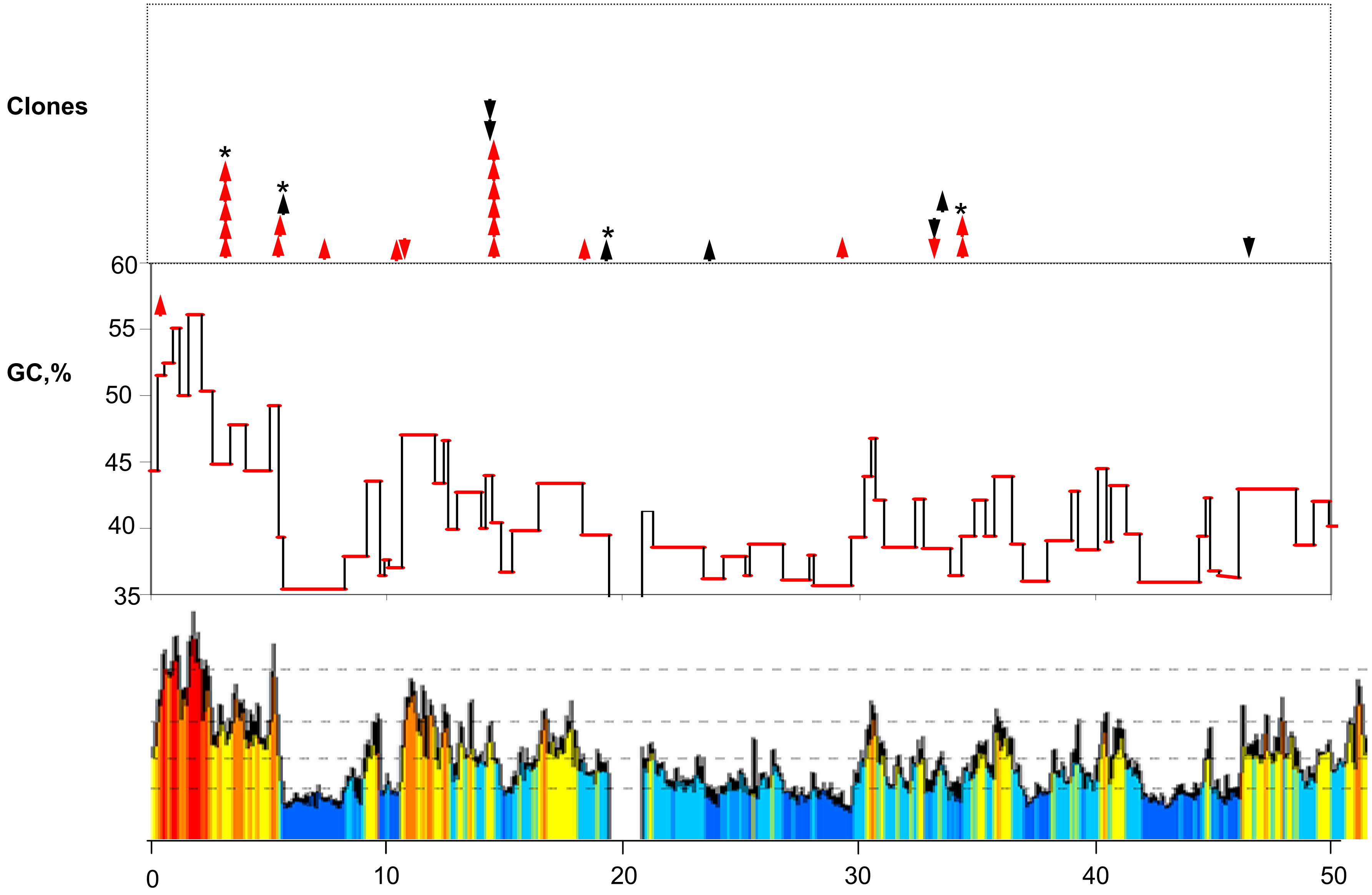

Chr4b

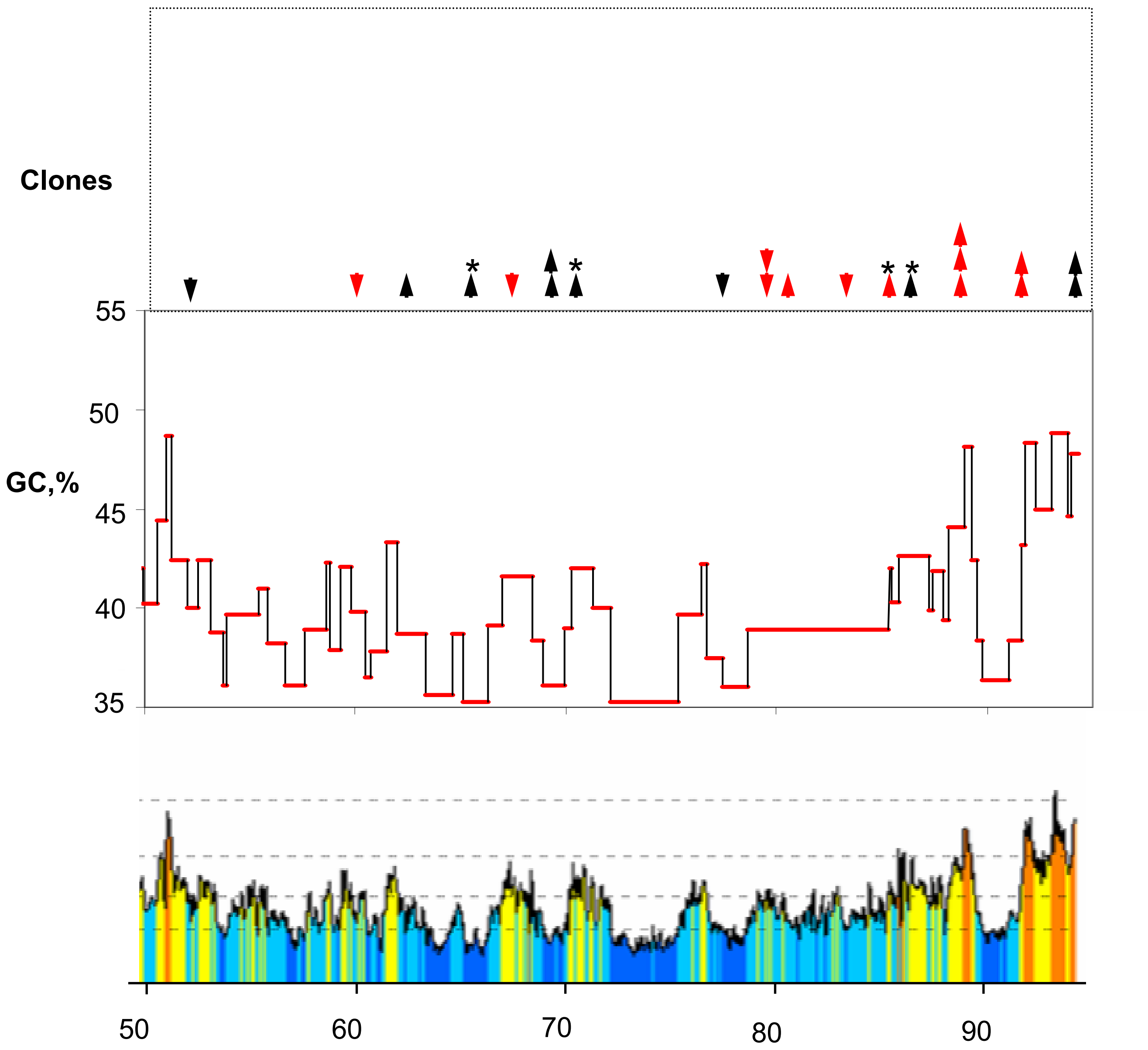

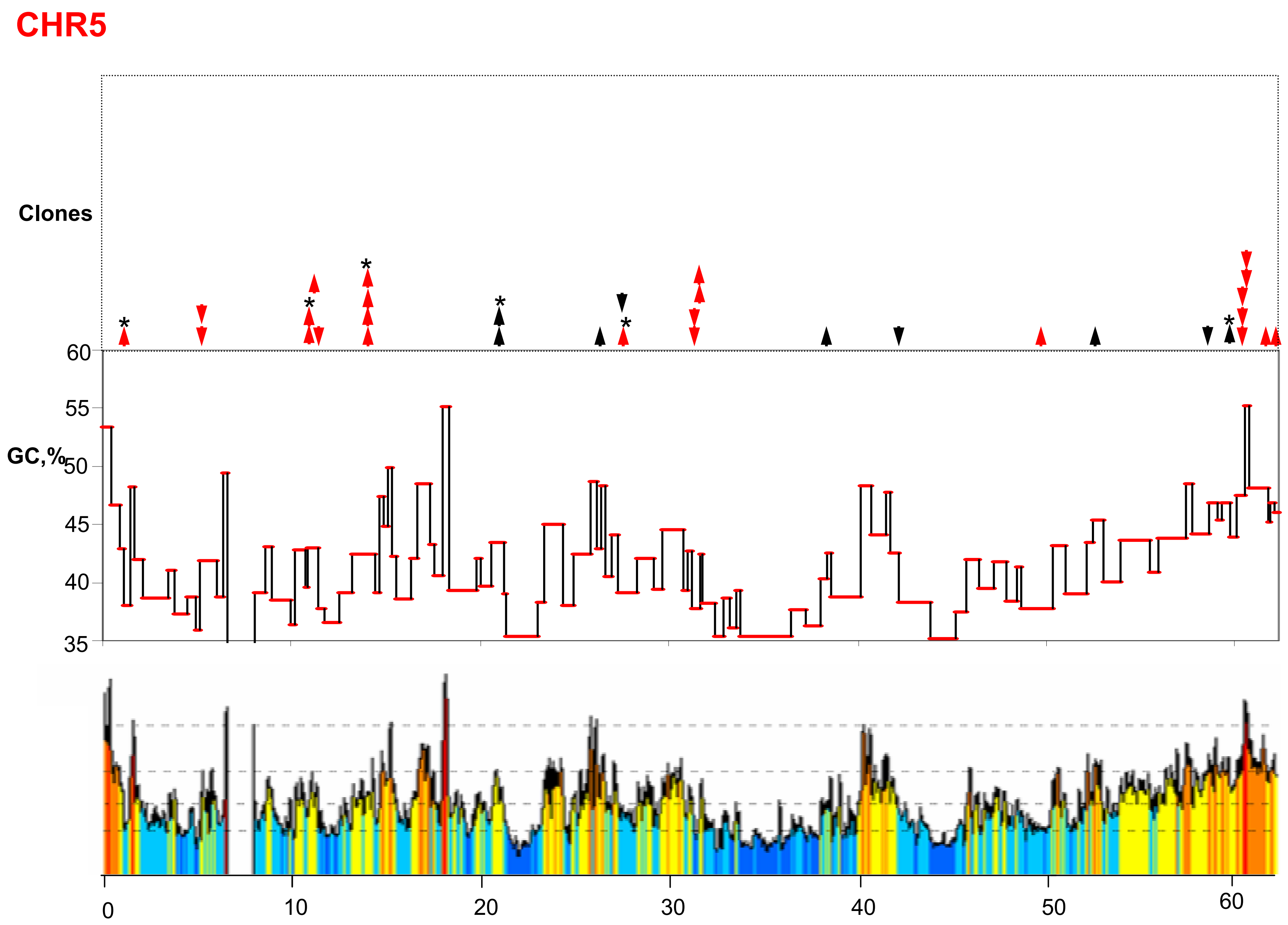
CHR5
Clones
GC,%
60
55
50
45
40
35
0
10
20
30
40
50
60

CHR6

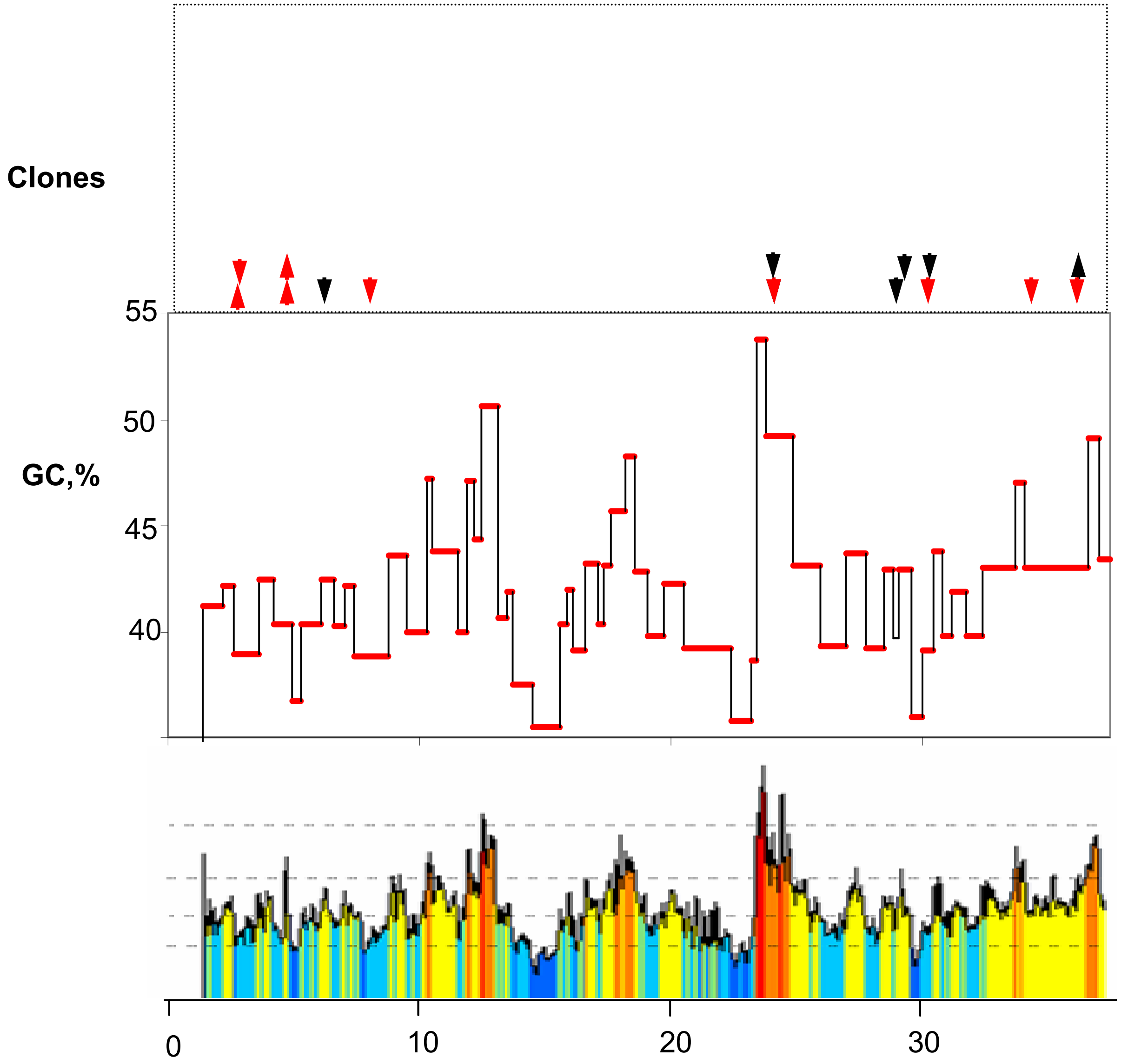

Chr7

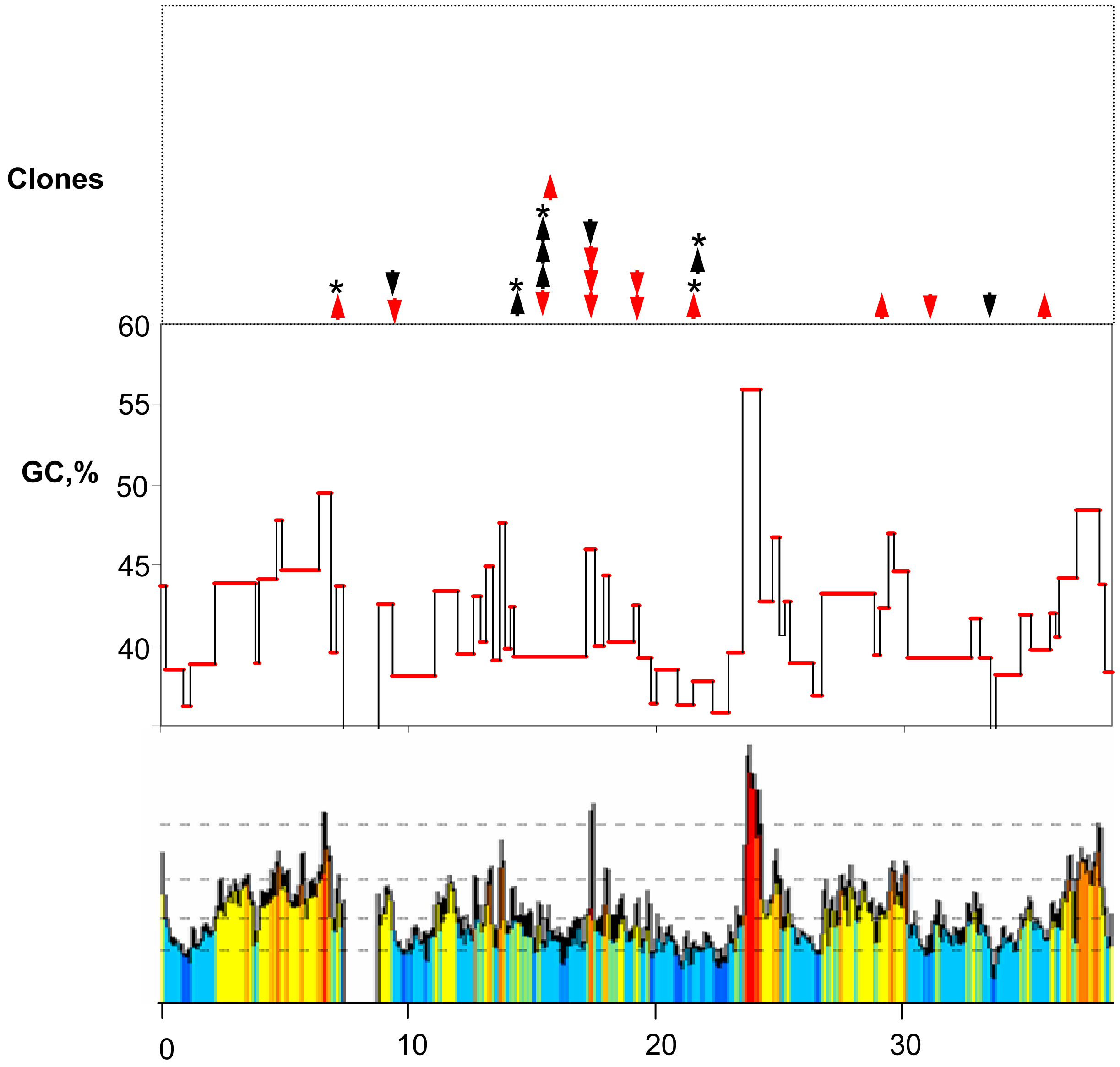

CHR8

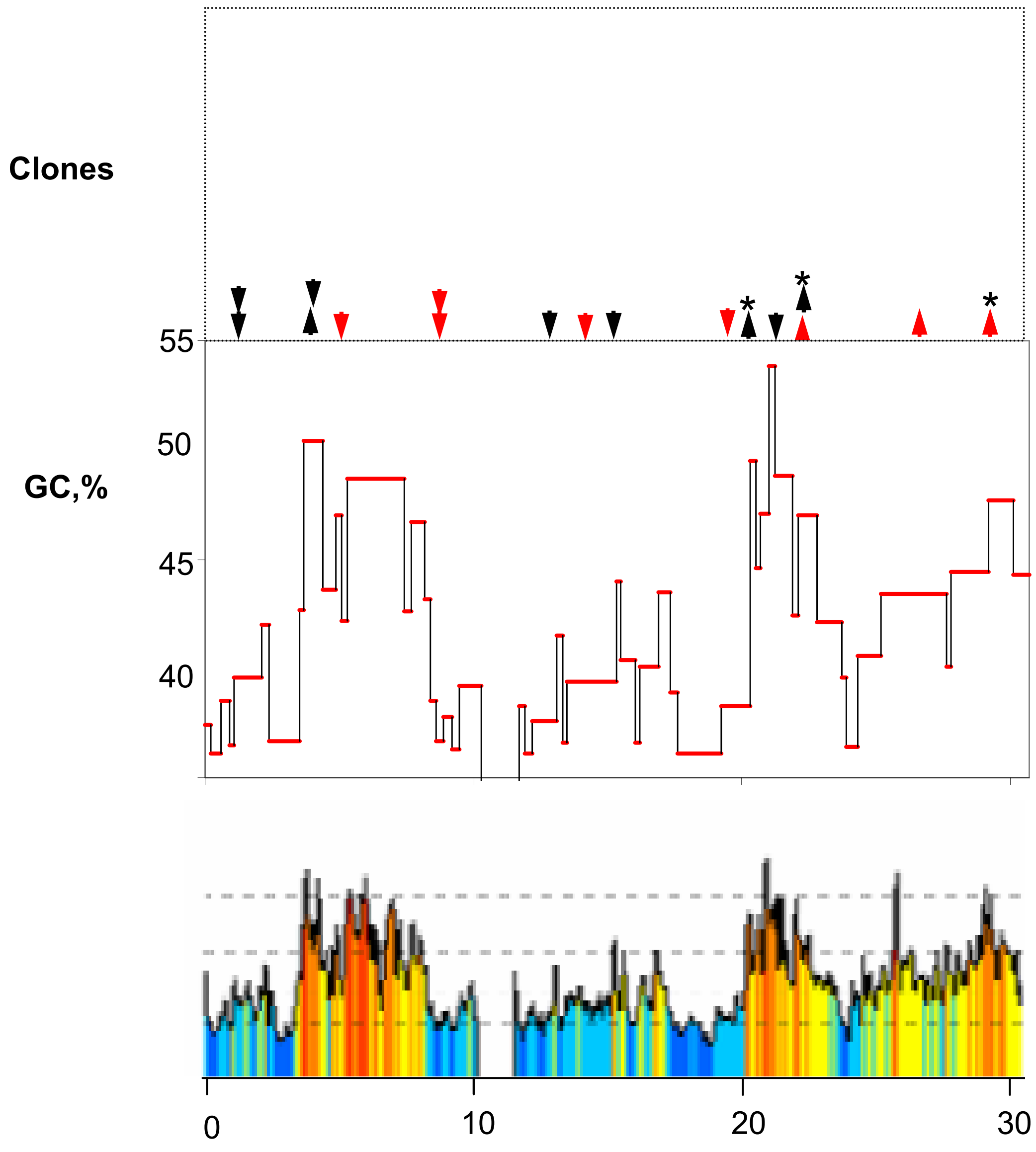

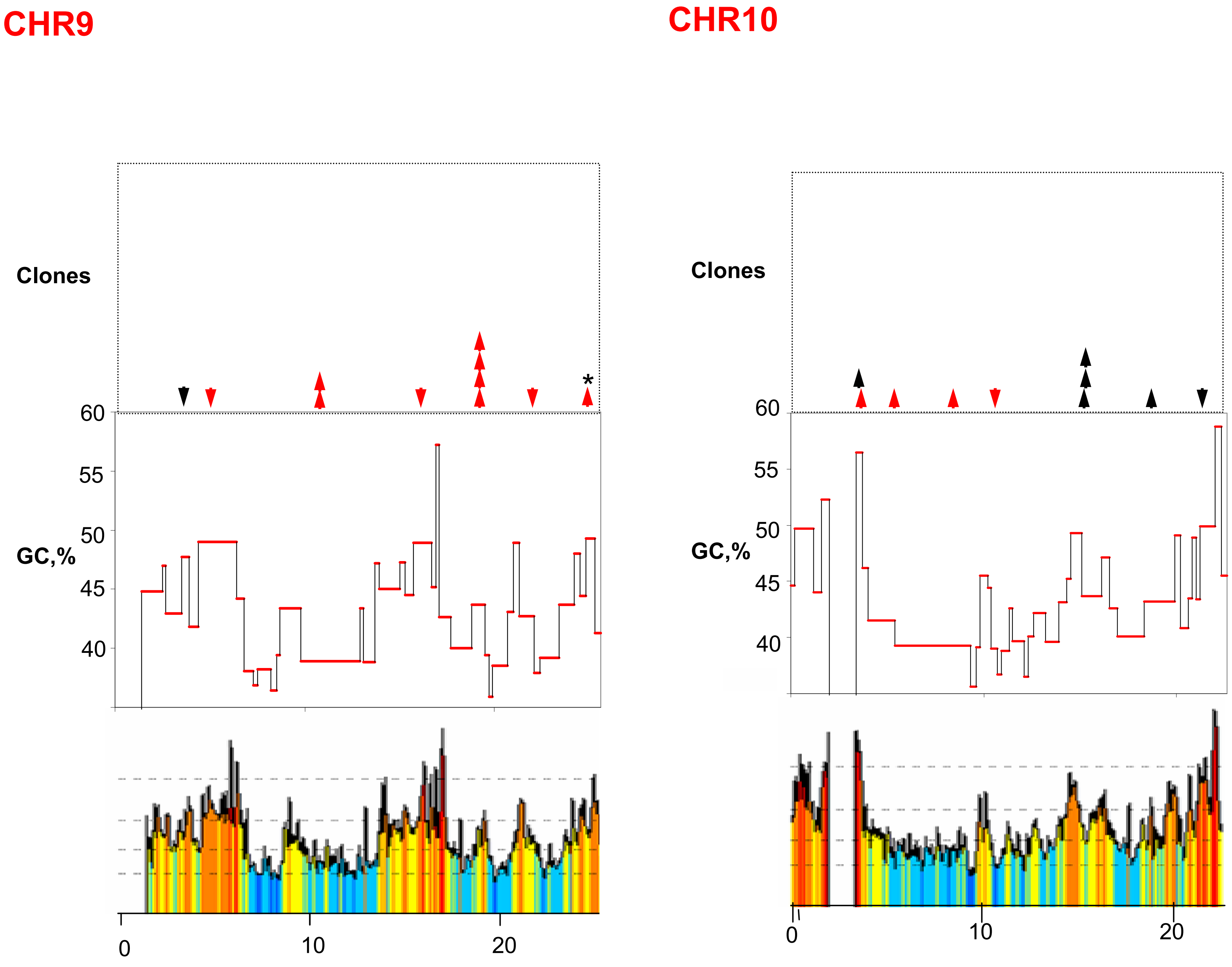

CHR9
CHR10
Clones
GC,%
60
55
50
45
40
0
10
20
*

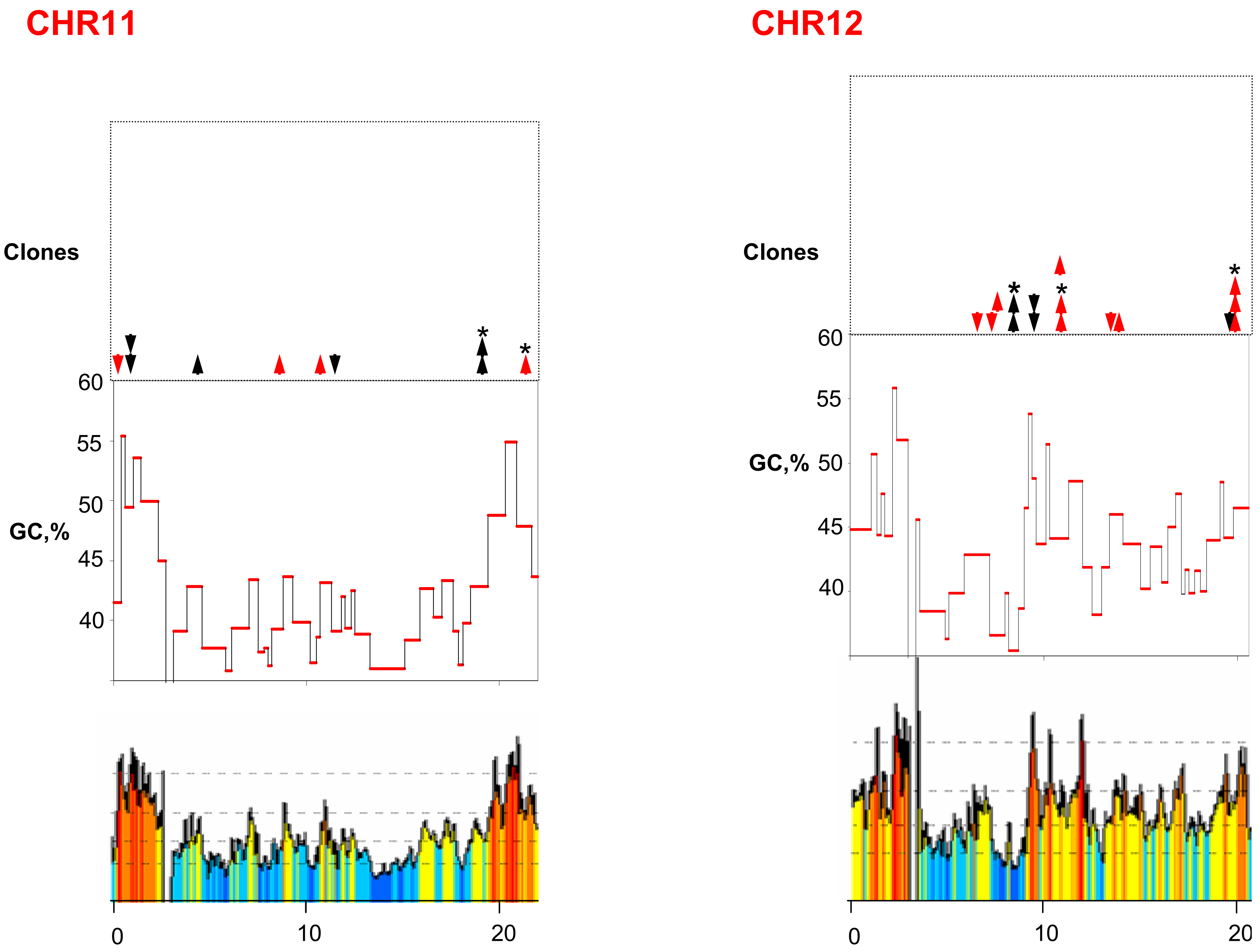

CHR11
Clones
60
55
50
GC,%
45
40
0
10
20
CHR12
Clones
60
55
GC,% 50
45
40
0
10
20

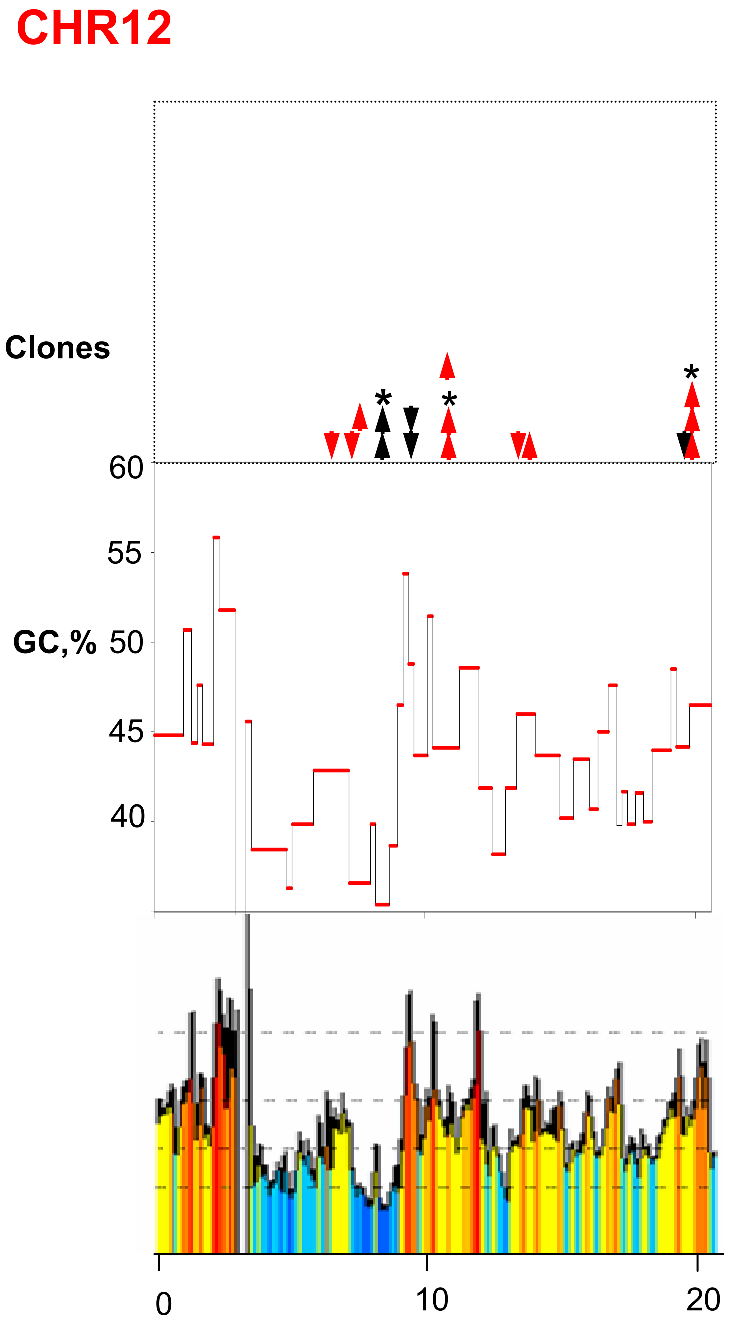

CHR12
Clones
GC,%
60
55
50
45
40
0
10
20

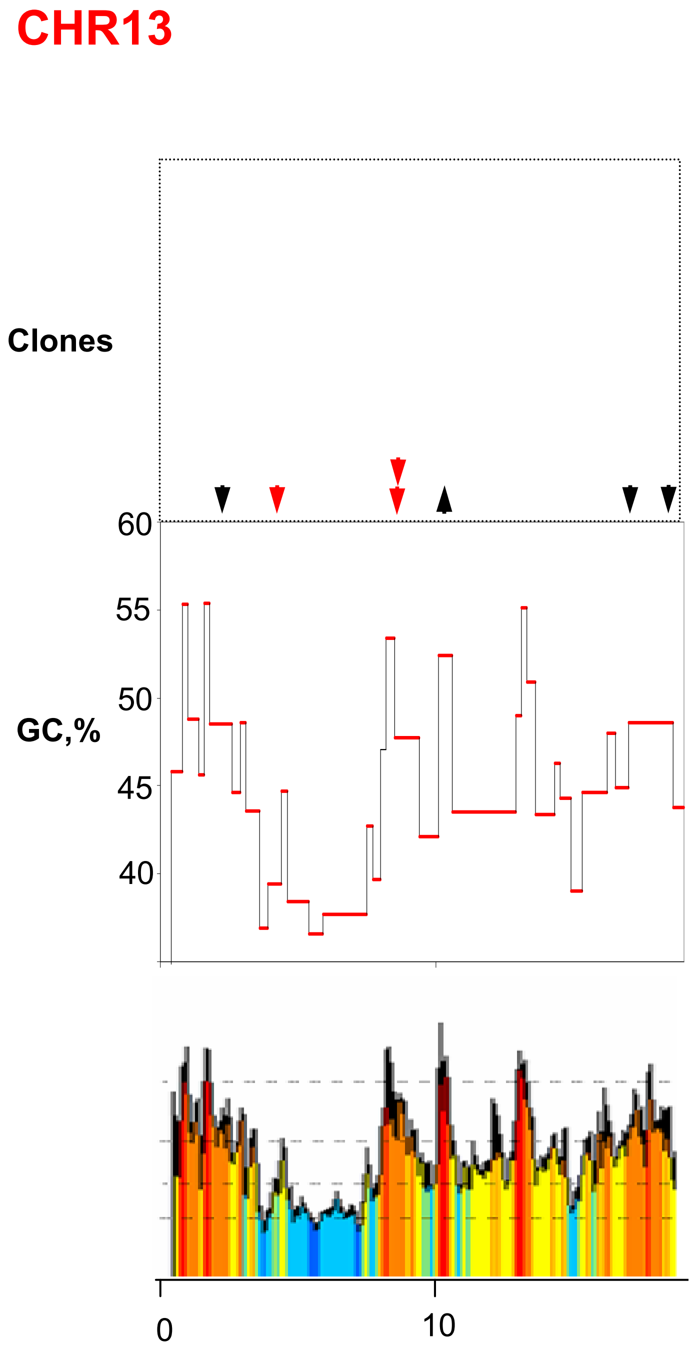

CHR13
Clones
GC,%
60
55
50
45
40
0
10

CHR 14

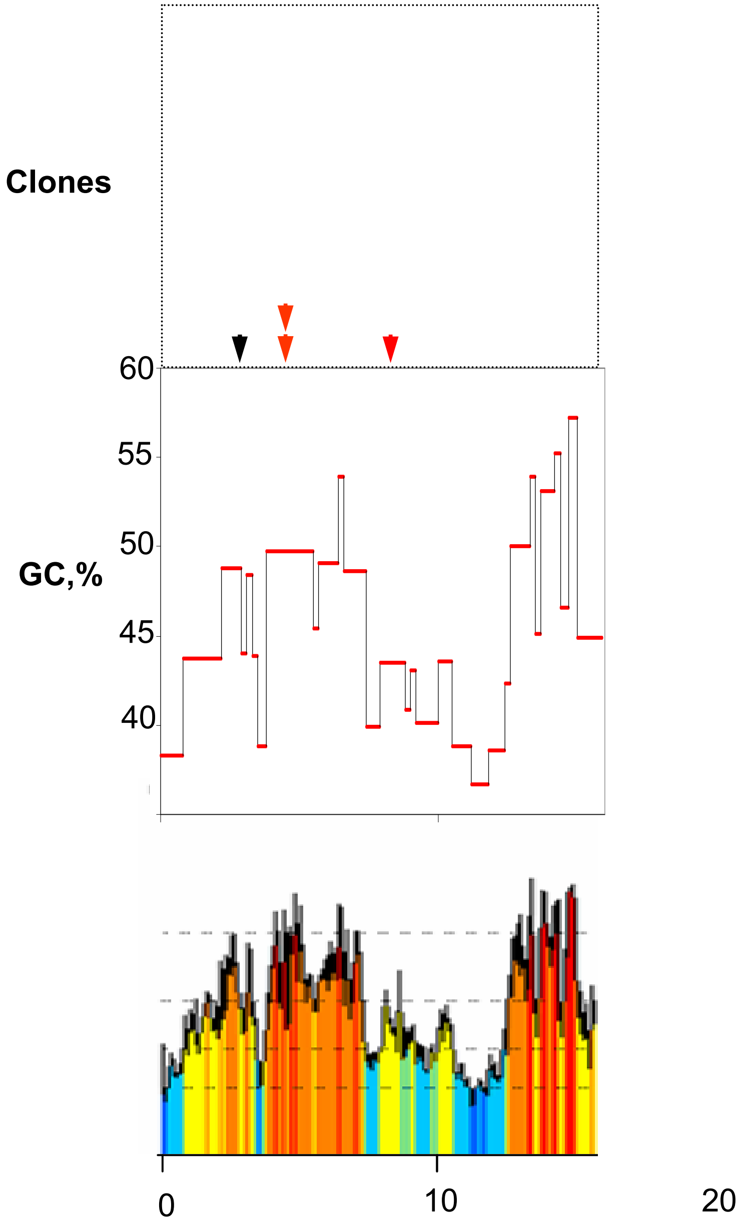

CHR15

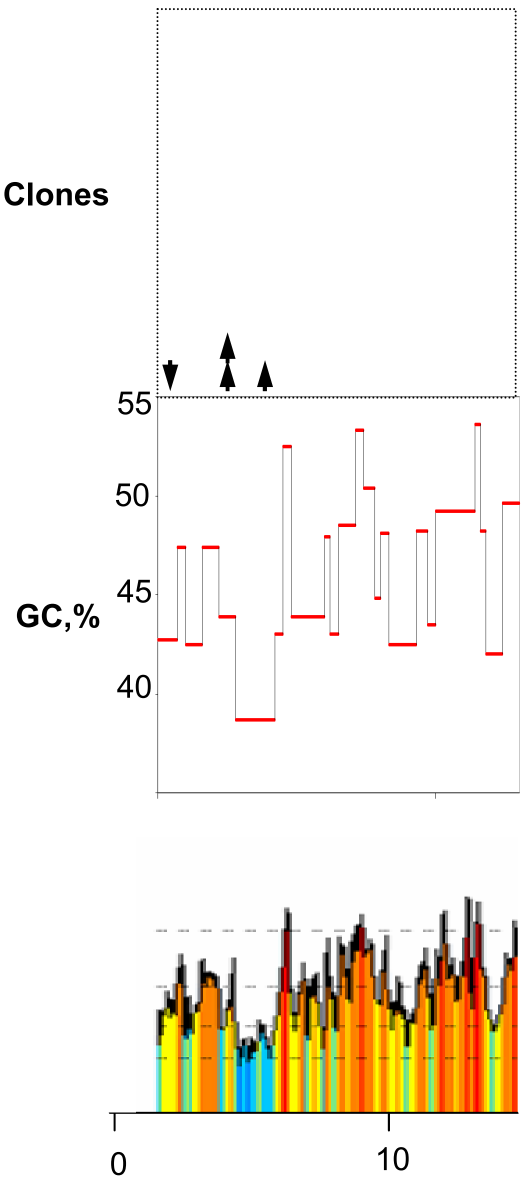

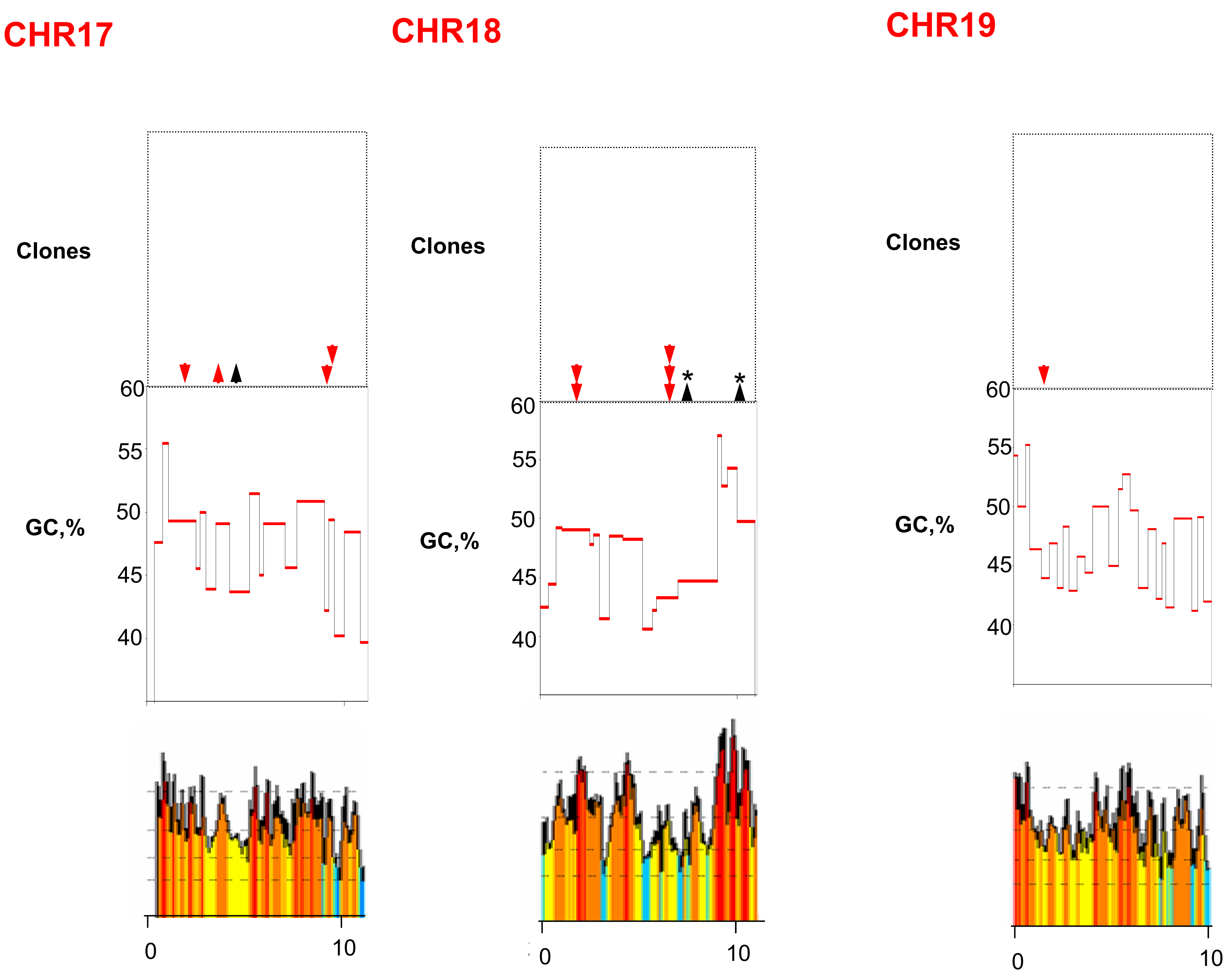

CHR17
CHR18
CHR19
Clones
Clones
Clones
GC,%
GC,%
GC,%
60
55
50
45
40
0
10

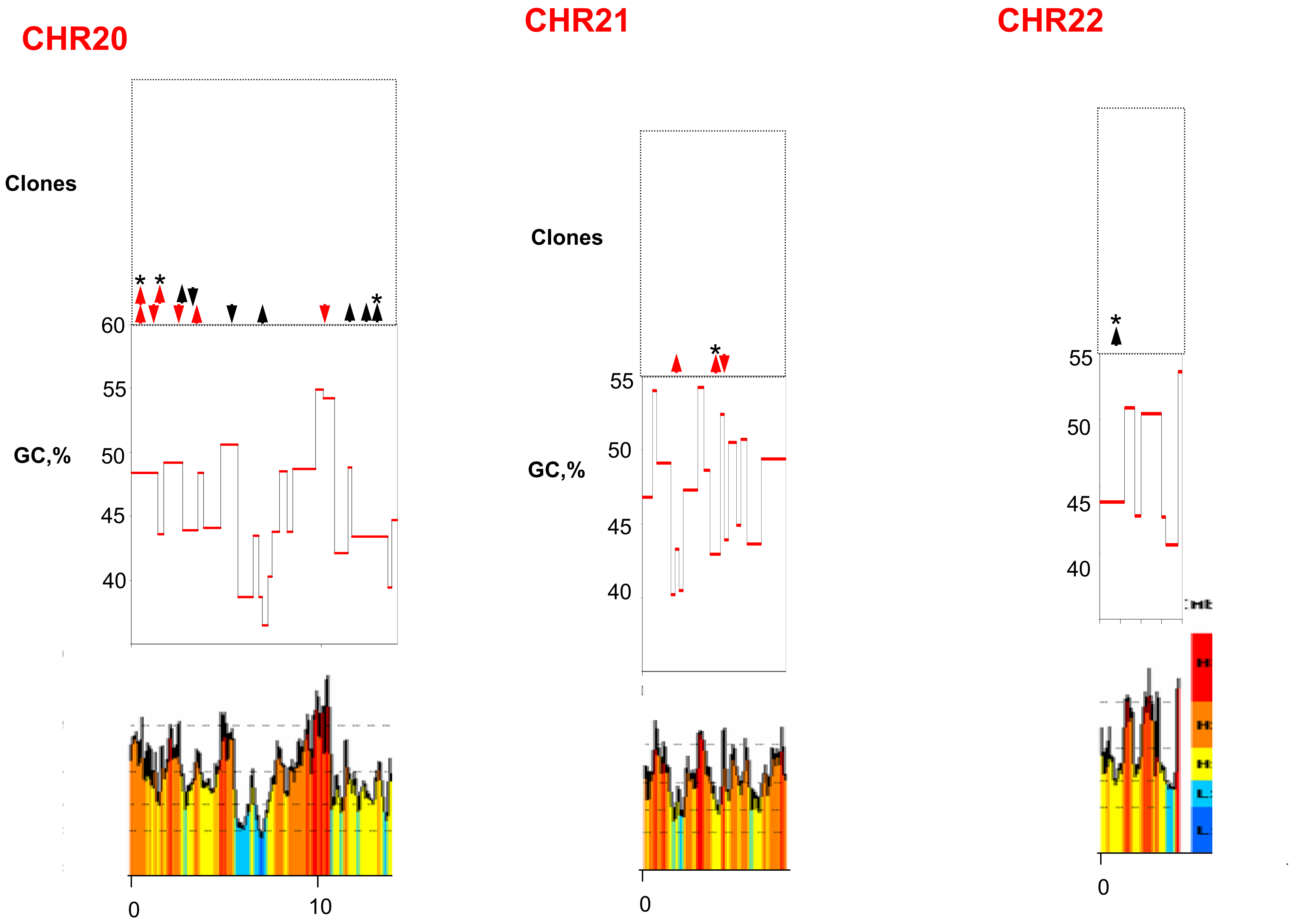

CHR20
CHR21
CHR22
Clones
Clones
GC,%
GC,%
60
55
50
45
40
55
50
45
40
55
50
45
40
0
10
0
0

CHR23

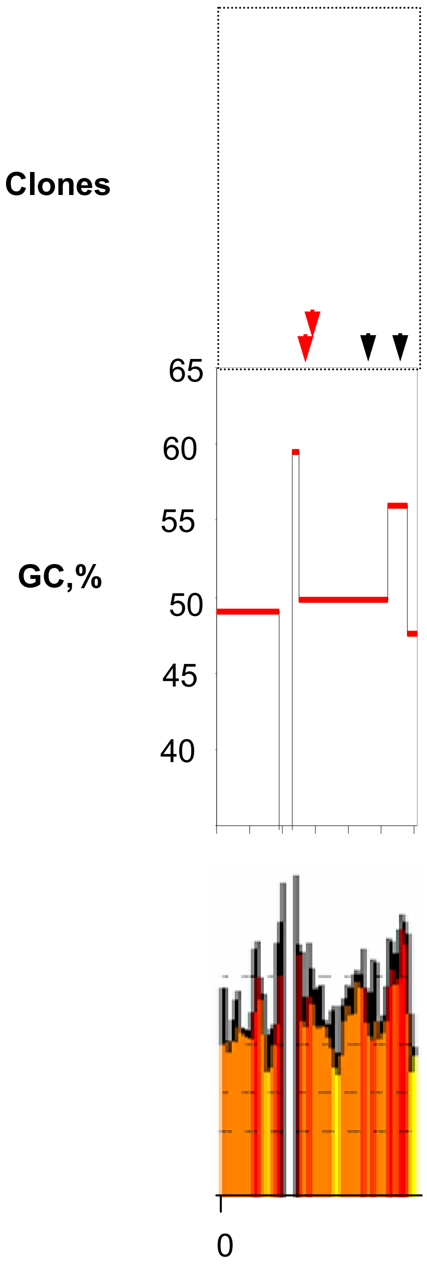

CHR24

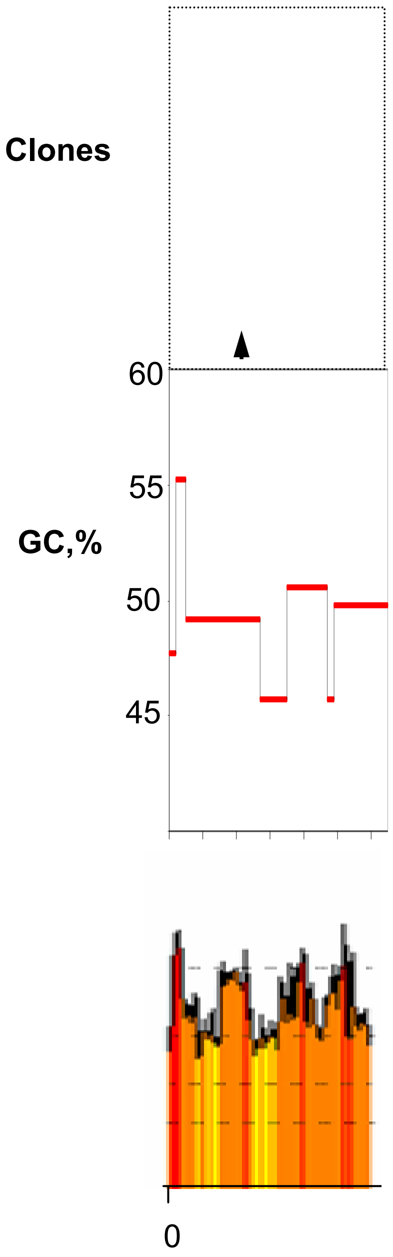

CHR25

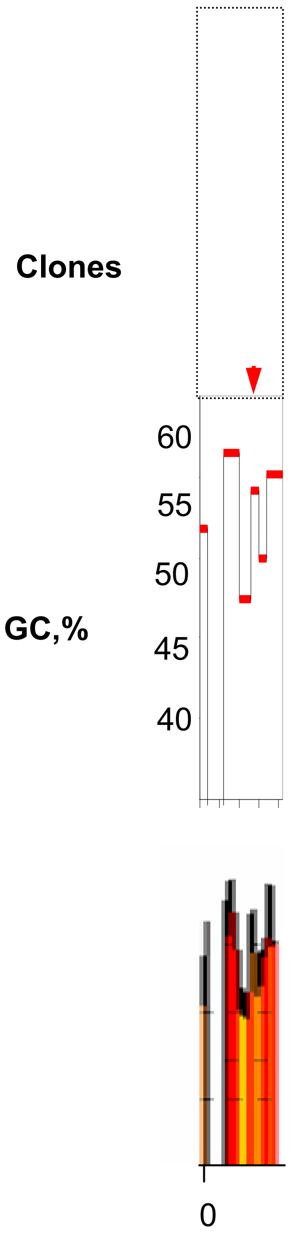

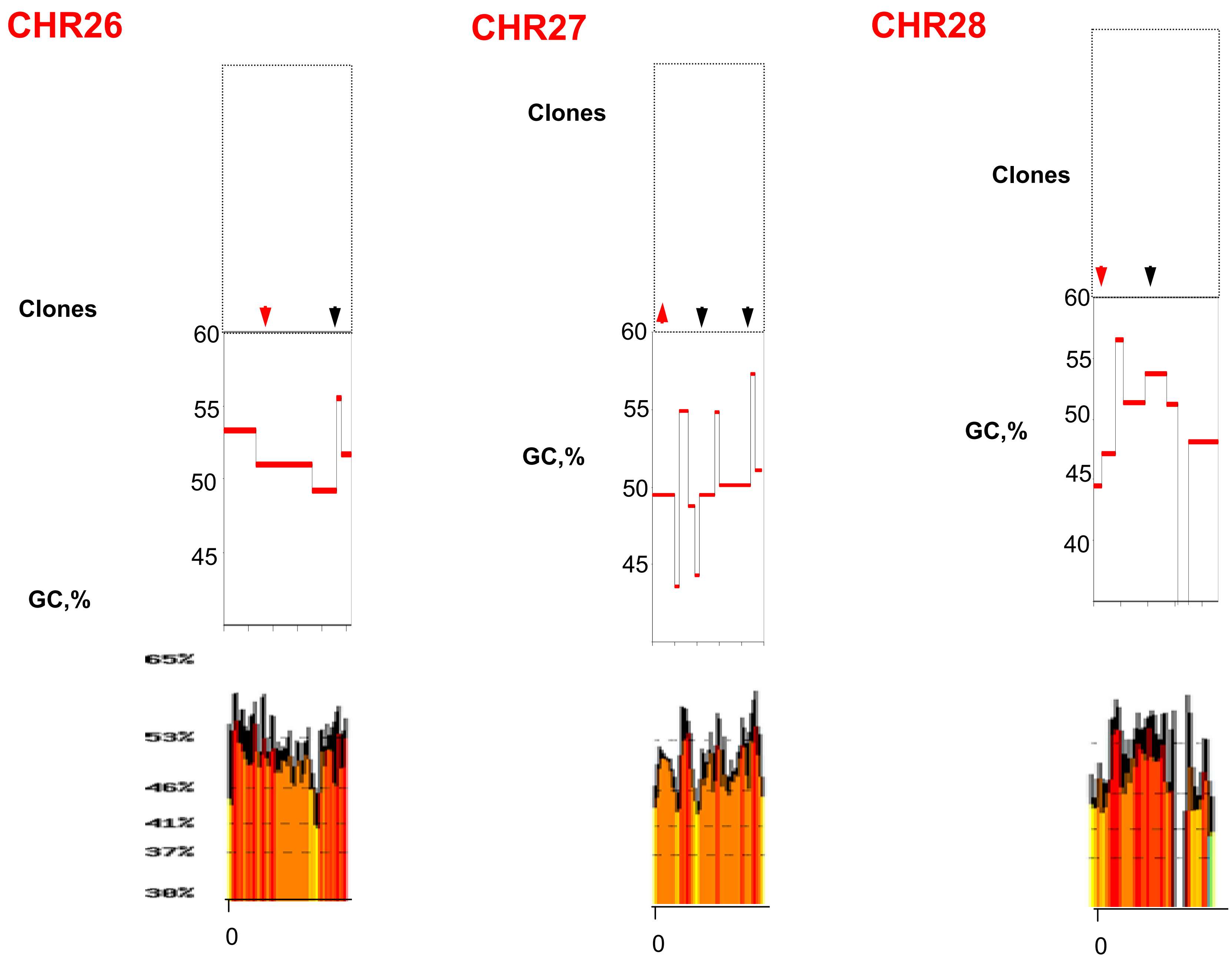
CHR26
Clones
60
55
50
45
GC,%
65%
53%
46%
41%
37%
30%
0
CHR27
Clones
60
55
GC,%
50
45
0
CHR28
Clones
60
55
50
GC,%
45
40
0

CHR Z

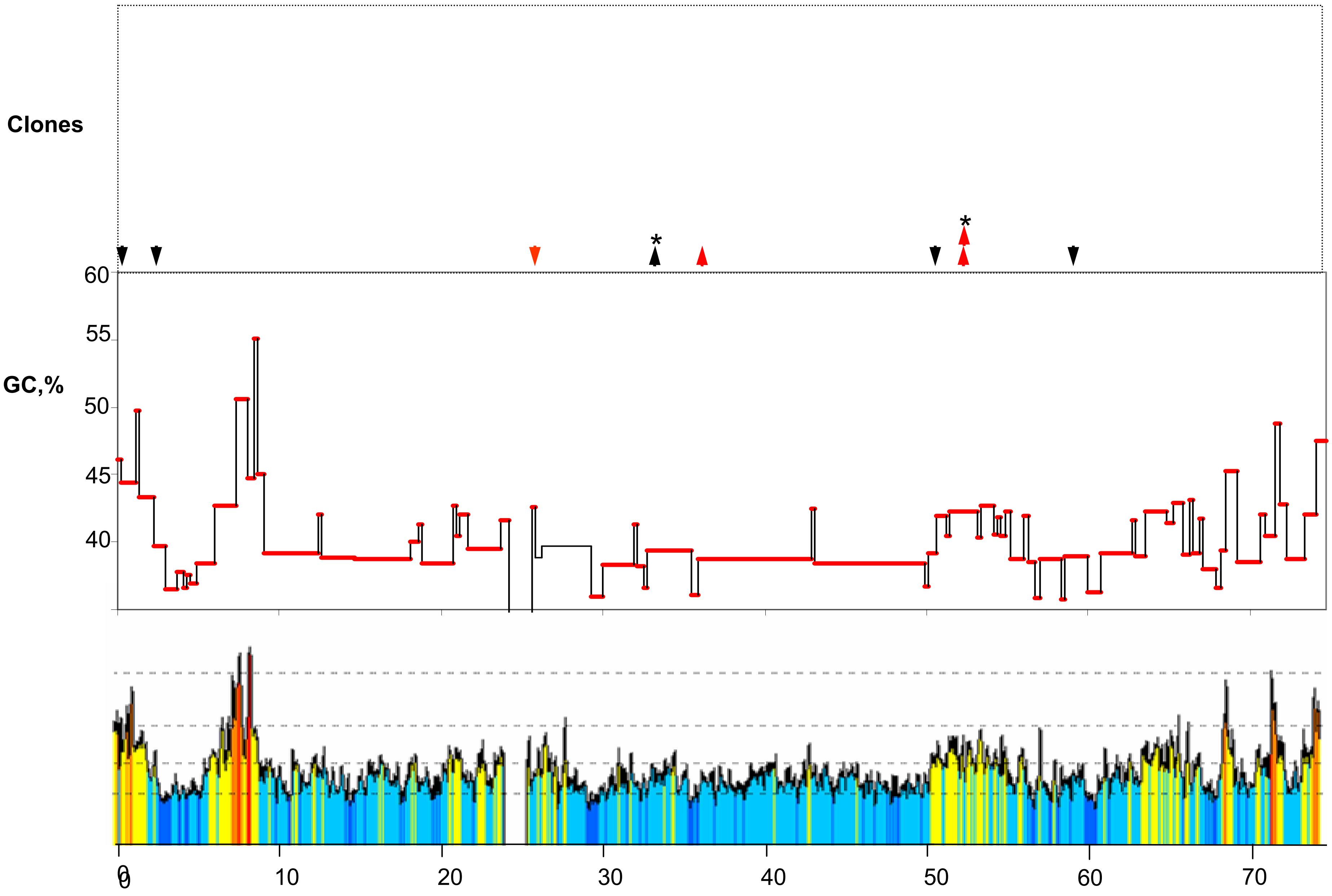

Supplement: Figure S1 — Clone density on chicken chromosomes. The chromosome location for all clones, for (A) autolysis and MNase digestion (B) respectively, was obtained by blast searching, using the URL: http://www.genome.ucsc.edu. The clone density was calculated as frequency of clones for chromosome / length of chromosome. A higher density of clones was found on microchromosomes. (7.86 MB PDF) [file pone.0005010.s001.pdf]
