# Supplementary figures and images for: The Early Apoptotic DNA Fragmentation Targets a Small Number of Specific Open Chromatin Regions
Source: PLoS One. 2009 Apr 6;4(4):e5010. doi: 10.1371/journal.pone.0005010 (PMC2661134; doi:10.1371/journal.pone.0005010)

Figure S2

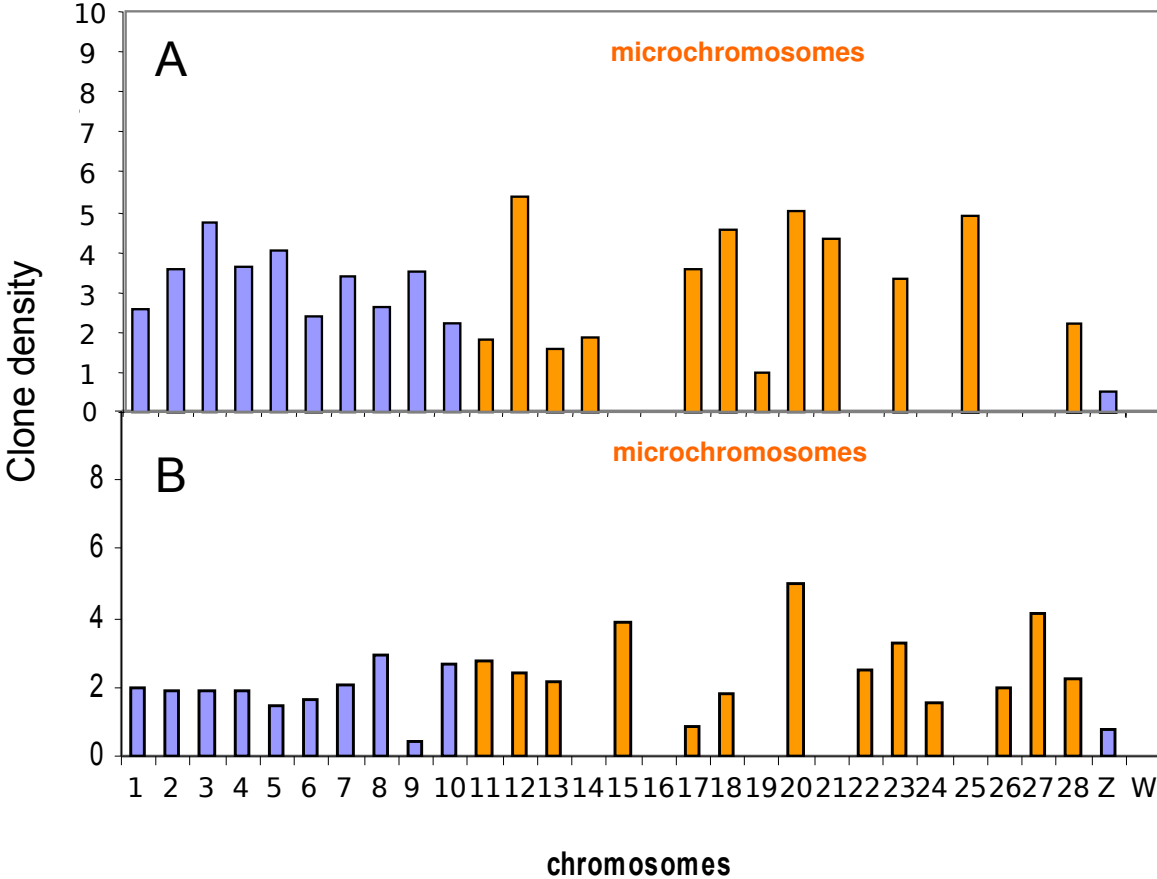

Supplement: Figure S2 — Mapping of apoptotic and MNase cuts on chicken chromosomes. The cut location, gene density and the GC level in the isochores (as in the Figure 3) are shown on all chicken choromosomes sequenced. (0.14 MB PDF) [file pone.0005010.s002.pdf]

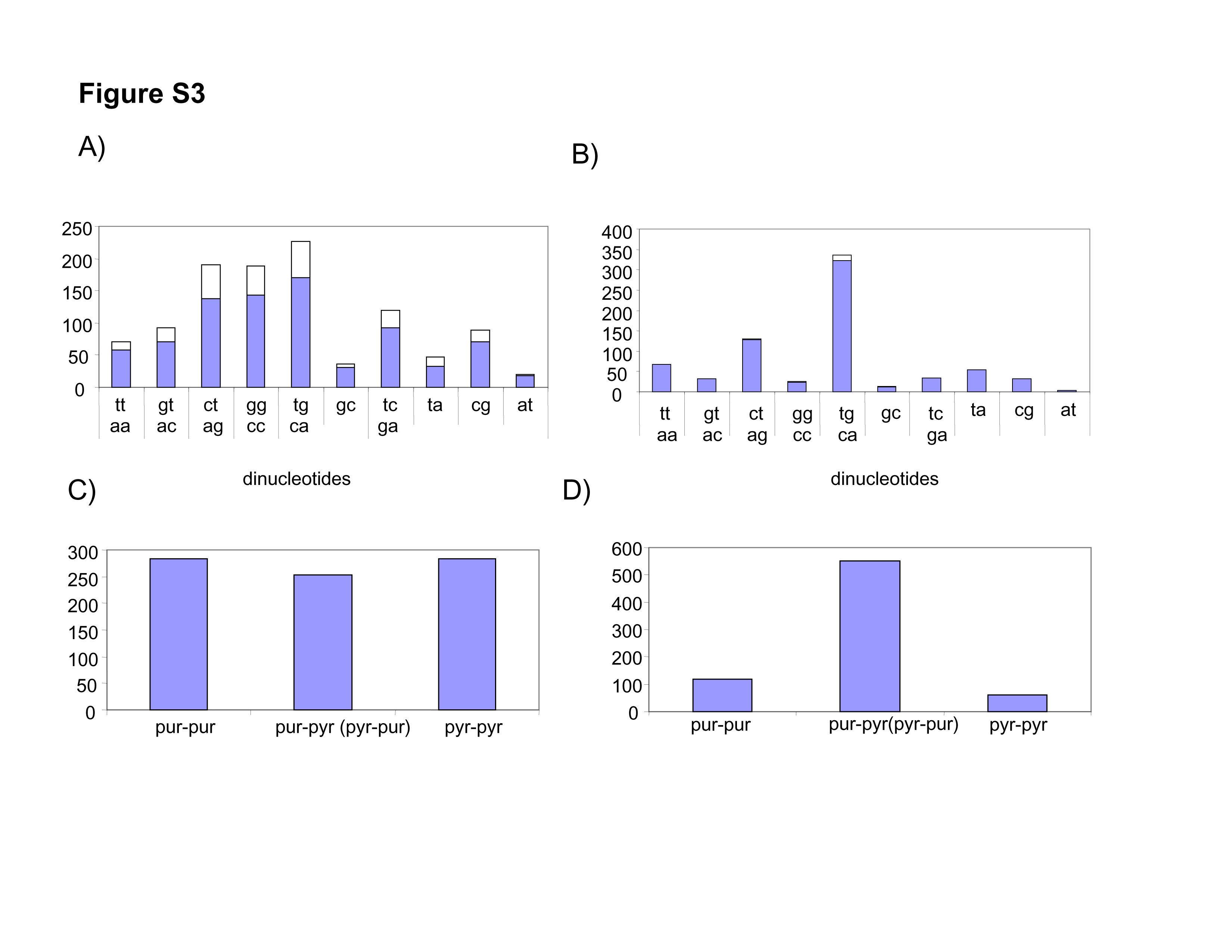

Supplement: Figure S3 — Terminal dinucleotides analysis. Frequencies of the terminal dinucleotides of the autolytic sequences cloned after autolysis (A) and MNase digestion (C). The contribution of the clones (blue bars) and of redundant clones (white bars) in the dinucleotides were plotted together for each dinucleotide. All pairs of terminal dinucleotides after autolysis (B) and MNase digestion (D) are classified in the three possible groups: purine/purine (pur/pur), pyrimidine/pyrimidine (pyr/pyr), and purine/pyrimidine or viceversa (pyr/pur). (0.69 MB TIF) [file pone.0005010.s003.tif]
