# Supplementary material for: The Early Apoptotic DNA Fragmentation Targets a Small Number of Specific Open Chromatin Regions
Source: PLoS One. 2009 Apr 6;4(4):e5010. doi: 10.1371/journal.pone.0005010 (PMC2661134; doi:10.1371/journal.pone.0005010)
Supplement: Table S1 — (0.36 MB DOC) [file pone.0005010.s004.doc]

**Table S1**

**The number and the chromosome location of the clones retrieved by blast search against UCSC genome browser, for both Autolysis and MNase digestion are reported.**

|  |  |  |  |  |  |
| --- | --- | --- | --- | --- | --- |
|  |  |  |  |  |  |
|  |  | **Autolysis** |  | **MNase** |  |
|  | **clone** | ***chromosome location** | **clone** | ***chromosome location** |  |
|  | 1 | chr1:104,460,895-104,461,040 | 1 | chr1:105,212,596-105,212,803 |  |
|  | 2 | chr1:125,120,137-125,120,327 | 2 | chr1:110,228,105-110,228,277 |  |
|  | 3 | chr1:131,600,406-131,600,606 | 3 | chr1:111,574,749-111,574,948 |  |
|  | 4 | chr1:144,759,099-144,759,410 | 4 | chr1:115,028,187-115,028,373 |  |
|  | 5 | chr1:149,474,294-149,474,454 | 5 | chr1:121,074,801-121,074,973 |  |
|  | 6 | chr1:150,638,598-150,638,766 | 6 | chr1:122,787,216-122,787,398 |  |
|  | 7 | chr1:164,747,134-164,747,578 | 7 | chr1:131,341,819-131,342,000 |  |
|  | 8 | chr1:170,258,426-170,258,603 | 8 | chr1:136,308,463-136,308,647 |  |
|  | 9 | chr1:174,254,678-174,254,857 | 9 | chr1:143,610,516-143,610,664 |  |
|  | 10 | chr1:175,702,952-175,703,138 | 10 | chr1:151,615,135-151,615,280 |  |
|  | 11 | chr1:175,702,968-175,703,081 | 11 | chr1:158,387,445-158,387,631 |  |
|  | 12 | chr1:180,742,380-180,742,517 | 12 | chr1:170,231,806-170,231,981 |  |
|  | 13 | chr1:189,746,734-189,746,885 | 13 | chr1:172,525,779-172,525,958 |  |
|  | 14 | chr1:191,336,677-191,336,824 | 14 | chr1:175,804,712-175,804,885 |  |
|  | 15 | chr1:2,552,557-2,552,710 | 15 | chr1:180,218,448-180,218,649 |  |
|  | 16 | chr1:200,150,631-200,151,005 | 16 | chr1:184,651,150-184,651,307 |  |
|  | 17 | chr1:21,126,934-21,127,094 | 17 | chr1:184,655,582-184,655,766 |  |
|  | 18 | chr1:29,408,411-29,408,599 | 18 | chr1:189,405,216-189,405,386 |  |
|  | 19 | chr1:31,501,464-31,501,610 | 19 | chr1:200,387,025-200,387,199 |  |
|  | 20 | chr1:35,887,163-35,887,294 | 20 | chr1:24,315,316-24,315,444 |  |
|  | 21 | chr1:36,127,233-36,127,403 | 21 | chr1:3,218,520-3,218,667 |  |
|  | 22 | chr1:41,155,683-41,155,802 | 22 | chr1:30,466,821-30,466,988 |  |
|  | 23 | chr1:58,603,981-58,604,155 | 23 | chr1:32,462,919-32,463,312 |  |
|  | 24 | chr1:6,089,282-6,089,447 | 24 | chr1:38,929,740-38,929,901 |  |
|  | 25 | chr1:62,436,991-62,437,156 | 25 | chr1:56,272,665-56,272,848 |  |
|  | 26 | chr1:62,997,866-62,998,034 | 26 | chr1:63,939,753-63,939,946 |  |
|  | 27 | chr1:63,638,114-63,638,279 | 27 | chr1:69,228,714-69,228,879 |  |
|  | 28 | chr1:73,019,012-73,019,192 | 28 | chr1:83,951,930-83,952,112 |  |
|  | 29 | chr1:76,797,103-76,797,262 | 29 | chr1:83,953,921-83,954,112 |  |
|  | 30 | chr1:99,896,991-99,897,113 | 30 | chr1:84,493,788-84,493,975 |  |
|  | 31 | chr10:10,990,255-10,990,426 | 31 | chr1:9,202,145-9,202,292 |  |
|  | 32 | chr10:12,518,800-12,518,994 | 32 | chr1:93,498,826-93,499,013 |  |
|  | 33 | chr10:3,952,586-3,952,729 | 33 | chr1:97,035,269-97,035,460 |  |
|  | 34 | chr10:5,312,328-5,312,521 | 34 | chr10:15,307,574-15,307,750 |  |
|  | 35 | chr10:8,654,456-8,654,602 | 35 | chr10:15,502,967-15,503,175 |  |
|  | 36 | chr11:10,915,808-10,915,907 | 36 | chr10:15,551,110-15,551,291 |  |
|  | 37 | chr11:21,913,557-21,913,748 | 37 | chr10:18,347,395-18,347,745 |  |
|  | 38 | chr11:429,359-429,506 | 38 | chr10:21,316,651-21,316,927 |  |
|  | 39 | chr11:8,869,412-8,869,586 | 39 | chr10:3,807,845-3,808,012 |  |
|  | 40 | chr12:10,815,980-10,816,199 | 40 | chr11:1,818,225-1,818,548 |  |
|  | 41 | chr12:13,086,673-13,086,968 | 41 | chr11:11,931,567-11,931,889 |  |
|  | 42 | chr12:19,873,999-19,874,153 | 42 | chr11:19,417,577-19,417,778 |  |
|  | 43 | chr12:6,718,732-6,718,857 | 43 | chr11:4,726,650-4,726,809 |  |
|  | 44 | chr12:7,082,476-7,082,737 | 44 | chr12:19,170,256-19,170,424 |  |
|  | 45 | chr12:7,536,227-7,536,386 | 45 | chr12:8,111,095-8,111,426 |  |
|  | 46 | chr13:4,845,325-4,845,568 | 46 | chr12:9,126,082-9,126,421 |  |
|  | 47 | chr13:8,843,957-8,844,035 | 47 | chr13:10,596,737-10,596,900 |  |
|  | 48 | chr1:30,778,288-30,778,437 | 48 | chr13:17,082,244-17,082,410 |  |
|  | 49 | chr14:4,318,196-4,318,321 | 49 | chr13:18,774,994-18,775,167 |  |
|  | 50 | chr14:8,239,748-8,239,926 | 50 | chr13:2,992,722-2,992,922 |  |
|  | 51 | chr17:2,037,150-2,037,262 | 51 | chr14:2,962,887-2,963,115 |  |
|  | 52 | chr17:3,271,513-3,271,648 | 52 | chr15:2,543,197-2,543,385 |  |
|  | 53 | chr17:9,049,433-9,049,575 | 53 | chr15:3,850,416-3,850,587 |  |
|  | 54 | chr17:9,281,582-9,281,771 | 54 | chr15:339,678-340,038 |  |
|  | 55 | chr18:1,189,540-1,189,745 | 55 | chr15:4,951,661-4,951,845 |  |
|  | 56 | chr18:6,768,003-6,768,184 | 56 | chr17:4,705,806-4,705,976 |  |
|  | 57 | chr19:1,428,353-1,428,478 | 57 | chr18:10,020,374-10,020,556 |  |
|  | 58 | chr2:1,274,399-1,274,561 | 58 | chr18:7,315,780-7,316,089 |  |
|  | 59 | chr2:100,215,536-100,215,709 | 59 | chr2:10,351,722-10,351,884 |  |
|  | 60 | chr2:100,607,137-100,607,340 | 60 | chr2:102,118,863-102,119,043 |  |
|  | 61 | chr2:105,518,256-105,518,409 | 61 | chr2:107,315,958-107,316,125 |  |
|  | 62 | chr2:108,653,555-108,653,741 | 62 | chr2:108,573,759-108,573,910 |  |
|  | 63 | chr2:109,493,525-109,493,669 | 63 | chr2:14,794,231-14,794,403 |  |
|  | 64 | chr2:121,105,240-121,105,387 | 64 | chr2:140,279,578-140,279,746 |  |
|  | 65 | chr2:125,019,120-125,019,289 | 65 | chr2:141,496,180-141,496,327 |  |
|  | 66 | chr2:130,066,637-130,066,816 | 66 | chr2:142,435,920-142,436,097 |  |
|  | 67 | chr2:130,293,180-130,293,368 | 67 | chr2:143,730,976-143,731,151 |  |
|  | 68 | chr2:131,201,369-131,201,528 | 68 | chr2:150,448,009-150,448,201 |  |
|  | 69 | chr2:142,464,534-142,464,721 | 69 | chr2:152,238,276-152,238,463 |  |
|  | 70 | chr2:154,548,958-154,549,103 | 70 | chr2:154,113,184-154,113,363 |  |
|  | 71 | chr2:24,579,096-24,579,260 | 71 | chr2:17,253,163-17,253,319 |  |
|  | 72 | chr2:28,074,613-28,075,131 | 72 | chr2:20,017,192-20,017,368 |  |
|  | 73 | chr2:3,513,227-3,513,354 | 73 | chr2:30,152,524-30,152,667 |  |
|  | 74 | chr2:3,813,041-3,813,158 | 74 | chr2:31,596,525-31,596,693 |  |
|  | 75 | chr2:32,529,523-32,529,625 | 75 | chr2:31,988,607-31,988,773 |  |
|  | 76 | chr2:33,982,982-33,983,128 | 76 | chr2:36,318,496-36,318,647 |  |
|  | 77 | chr2:45,845,760-45,845,935 | 77 | chr2:60,192,247-60,212,417 |  |
|  | 78 | chr2:50,710,641-50,710,736 | 78 | chr2:60,202,247-60,202,416 |  |
|  | 79 | chr2:51,956,433-51,956,582 | 79 | chr2:66,670,675-66,670,831 |  |
|  | 80 | chr2:62,597,759-62,597,913 | 80 | chr2:80,134,172-80,134,346 |  |
|  | 81 | chr2:64,428,890-64,429,063 | 81 | chr2:82,391,122-82,391,284 |  |
|  | 82 | chr2:7,286,065-7,286,213 | 82 | chr2:92,368,018-92,368,190 |  |
|  | 83 | chr2:85,170,108-85,170,643 | 83 | chr2:94,410,295-94,410,458 |  |
|  | 84 | chr2:87,126,955-87,127,135 | 84 | chr20:11,408,364-11,408,524 |  |
|  | 85 | chr2:90,388,453-90,388,625 | 85 | chr20:11,756,371-11,756,541 |  |
|  | 86 | chr2:91,088,430-91,088,597 | 86 | chr20:13,327,572-13,327,734 |  |
|  | 87 | chr2:92,721,825-92,721,966 | 87 | chr20:2,910,704-2,910,873 |  |
|  | 88 | chr2:93,377,267-93,378,032 | 88 | chr20:3,230,704-3,231,041 |  |
|  | 89 | chr2:94,436,817-94,437,002 | 89 | chr20:5,319,588-5,319,758 |  |
|  | 90 | chr2:95,505,560-95,505,765 | 90 | chr20:7,002,900-7,003,070 |  |
|  | 91 | chr20:1,130,868-1,131,020 | 91 | chr22:978,888-979,056 |  |
|  | 92 | chr20:1,429,593-1,429,751 | 92 | chr23:4,343,960-4,344,150 |  |
|  | 93 | chr20:10,844,338-10,844,505 | 93 | chr23:5,708,901-5,709,226 |  |
|  | 94 | chr20:2,596,480-2,596,736 | 94 | chr24:2,358,716-2,358,861 |  |
|  | 95 | chr20:3,425,578-3,425,721 | 95 | chr26:4,706,508-4,706,676 |  |
|  | 96 | chr20:347,515-347,669 | 96 | chr27:2,062,496-2,062,658 |  |
|  | 97 | chr21:1,193,115-1,193,263 | 97 | chr27:4,082,745-4,082,926 |  |
|  | 98 | chr21:3,135,741-3,135,872 | 98 | chr28:2,849,787-2,849,950 |  |
|  | 99 | chr21:399,722-400,091 | 99 | chr3:101,128,632-101,128,820 |  |
|  | 100 | chr23:2,658,241-2,658,395 | 100 | chr3:103,350,132-103,350,297 |  |
|  | 101 | chr23:2,753,644-2,753,805 | 101 | chr3:103,475,920-103,476,089 |  |
|  | 102 | chr25:1,275,996-1,276,440 | 102 | chr3:111,379,581-111,379,755 |  |
|  | 103 | chr26:1,170,844-1,171,248 | 103 | chr3:36,337,947-36,338,103 |  |
|  | 104 | chr27:350,873-351,052 | 104 | chr3:36,757,893-36,758,055 |  |
|  | 105 | chr28:268,566-268,724 | 105 | chr3:44,590,473-44,590,644 |  |
|  | 106 | chr3:109,555,455-109,555,613 | 106 | chr3:48,595,745-48,595,884 |  |
|  | 107 | chr3:14,650,698-14,650,892 | 107 | chr3:64,205,150-64,205,318 |  |
|  | 108 | chr3:15,339,102-15,339,283 | 108 | chr3:70,482,117-70,482,293 |  |
|  | 109 | chr3:18,262,124-18,262,254 | 109 | chr3:750,312-750,478 |  |
|  | 110 | chr3:24,882,433-24,882,608 | 110 | chr3:80,081,392-80,081,560 |  |
|  | 111 | chr3:29,027,477-29,027,643 | 111 | chr3:80,962,832-80,963,158 |  |
|  | 112 | chr3:32,015,697-32,015,770 | 112 | chr3:81,122,463-81,122,625 |  |
|  | 113 | chr3:32,121,169-32,121,305 | 113 | chr3:83,485,562-83,485,728 |  |
|  | 114 | chr3:39,822,983-39,823,137 | 114 | chr3:91,886,740-91,886,911 |  |
|  | 115 | chr3:46,003,643-46,003,804 | 115 | chr3:92,334,502-92,334,684 |  |
|  | 116 | chr3:47,002,048-47,002,241 | 116 | chr4:14,354,558-14,354,705 |  |
|  | 117 | chr3:49,818,952-49,819,158 | 117 | chr4:19,224,492-19,224,671 |  |
|  | 118 | chr3:50,043,047-50,043,294 | 118 | chr4:23,814,850-23,815,029 |  |
|  | 119 | chr3:53,963,177-53,963,330 | 119 | chr4:33,353,870-33,354,050 |  |
|  | 120 | chr3:55,415,960-55,416,113 | 120 | chr4:33,796,867-33,797,030 |  |
|  | 121 | chr3:58,289,268-58,289,402 | 121 | chr4:46,700,766-46,700,926 |  |
|  | 122 | chr3:61,498,047-61,498,207 | 122 | chr4:5,789,218-5,789,395 |  |
|  | 123 | chr3:63,913,737-63,913,906 | 123 | chr4:52,047,753-52,047,975 |  |
|  | 124 | chr3:65,670,355-65,670,651 | 124 | chr4:62,483,454-62,483,617 |  |
|  | 125 | chr3:67,696,790-67,697,236 | 125 | chr4:65,457,231-65,457,415 |  |
|  | 126 | chr3:67,876,714-67,876,878 | 126 | chr4:69,256,500-69,256,663 |  |
|  | 127 | chr3:7,560,400-7,560,579 | 127 | chr4:70,543,545-70,543,700 |  |
|  | 128 | chr3:71,629,413-71,629,600 | 128 | chr4:77,577,340-77,577,511 |  |
|  | 129 | chr3:8,754,117-8,754,262 | 129 | chr4:86,486,458-86,486,644 |  |
|  | 130 | chr3:83,436,560-83,436,729 | 130 | chr4:94,106,216-94,106,387 |  |
|  | 131 | chr3:9,589,474-9,589,654 | 131 | chr5:21,085,550-21,085,729 |  |
|  | 132 | chr4:10,345,742-10,345,915 | 132 | chr5:26,219,375-26,219,730 |  |
|  | 133 | chr4:10,951,137-10,951,306 | 133 | chr5:27,678,815-27,678,985 |  |
|  | 134 | chr4:14,617,181-14,617,326 | 134 | chr5:38,195,534-38,195,683 |  |
|  | 135 | chr4:18,014,280-18,014,405 | 135 | chr5:42,271,914-42,272,082 |  |
|  | 136 | chr4:29,143,525-29,144,026 | 136 | chr5:52,553,273-52,553,443 |  |
|  | 137 | chr4:3,156,737-3,156,914 | 137 | chr5:58,633,326-58,633,515 |  |
|  | 138 | chr4:33,134,455-33,134,650 | 138 | chr5:59,743,353-59,743,516 |  |
|  | 139 | chr4:34,616,109-34,616,280 | 139 | chr6:24,177,605-24,177,759 |  |
|  | 140 | chr4:48,964,228-48,964,382 | 140 | chr6:29,133,815-29,133,987 |  |
|  | 141 | chr4:5,551,331-5,551,513 | 141 | chr6:29,941,150-29,941,319 |  |
|  | 142 | chr4:60,337,412-60,337,582 | 142 | chr6:30,724,035-30,724,212 |  |
|  | 143 | chr4:67,425,334-67,425,479 | 143 | chr6:36,728,783-36,728,931 |  |
|  | 144 | chr4:7,370,021-7,370,159 | 144 | chr6:6,333,090-6,333,267 |  |
|  | 145 | chr4:79,666,308-79,666,491 | 145 | chr7:14,226,212-14,226,375 |  |
|  | 146 | chr4:80,744,401-80,744,543 | 146 | chr7:15,448,590-15,448,780 |  |
|  | 147 | chr4:83,528,004-83,528,180 | 147 | chr7:17,604,810-17,604,981 |  |
|  | 148 | chr4:85,480,177-85,480,400 | 148 | chr7:21,698,940-21,699,097 |  |
|  | 149 | chr4:88,963,447-88,963,552 | 149 | chr7:33,849,226-33,849,395 |  |
|  | 150 | chr4:88,964,206-88,964,341 | 150 | chr7:9,990,069-9,990,213 |  |
|  | 151 | chr4:91,846,737-91,846,865 | 151 | chr8:1,474,988-1,475,169 |  |
|  | 152 | chr5:1,466,764-1,466,923 | 152 | chr8:13,004,675-13,004,858 |  |
|  | 153 | chr5:11,066,409-11,066,853 | 153 | chr8:15,600,962-15,601,123 |  |
|  | 154 | chr5:11,344,229-11,344,359 | 154 | chr8:20,482,087-20,482,420 |  |
|  | 155 | chr5:11,610,291-11,610,457 | 155 | chr8:21,593,534-21,593,697 |  |
|  | 156 | chr5:14,057,568-14,057,731 | 156 | chr8:22,622,674-22,622,902 |  |
|  | 157 | chr5:27,720,973-27,721,455 | 157 | chr8:3,296,706-3,296,859 |  |
|  | 158 | chr5:31,321,093-31,321,411 | 158 | chr8:3,831,165-3,831,513 |  |
|  | 159 | chr5:31,629,504-31,629,677 | 159 | chr9:3,815,985-3,816,147 |  |
|  | 160 | chr5:49,897,643-49,897,799 | 160 | chrZ:2,455,135-2,455,304 |  |
|  | 161 | chr5:5,746,595-5,746,766 | 161 | chrZ:33,361,534-33,361,719 |  |
|  | 162 | chr5:51,605,569-51,605,739 | 162 | chrZ:387,828-388,104 |  |
|  | 163 | chr5:60,375,915-60,376,338 | 163 | chrZ:50,920,811-50,920,963 |  |
|  | 164 | chr5:60,581,876-60,582,051 | 164 | chrZ:59,193,090-59,193,257 |  |
|  | 165 | chr5:61,753,234-61,753,563 | 165 | chrZ:60,139,377-60,139,545 |  |
|  | 166 | chr5:62,148,262-62,148,401 |  |  |  |
|  | 167 | chr6:2,581,448-2,581,612 |  |  |  |
|  | 168 | chr6:2,817,092-2,817,174 |  |  |  |
|  | 169 | chr6:24,457,917-24,458,069 |  |  |  |
|  | 170 | chr6:30,564,604-30,564,730 |  |  |  |
|  | 171 | chr6:34,989,167-34,989,312 |  |  |  |
|  | 172 | chr6:36,642,794-36,642,987 |  |  |  |
|  | 173 | chr6:5,033,938-5,034,089 |  |  |  |
|  | 174 | chr6:8,197,641-8,197,781 |  |  |  |
|  | 175 | chr7:15,351,493-15,351,578 |  |  |  |
|  | 176 | chr7:15,810,418-15,810,666 |  |  |  |
|  | 177 | chr7:17,657,945-17,658,098 |  |  |  |
|  | 178 | chr7:19,248,969-19,249,122 |  |  |  |
|  | 179 | chr7:21,606,236-21,606,373 |  |  |  |
|  | 180 | chr7:29,021,470-29,021,676 |  |  |  |
|  | 181 | chr7:31,611,610-31,611,781 |  |  |  |
|  | 182 | chr7:35,733,545-35,733,713 |  |  |  |
|  | 183 | chr7:6,891,238-6,891,402 |  |  |  |
|  | 184 | chr7:9,234,612-9,234,790 |  |  |  |
|  | 185 | chr8:15,848,749-15,848,916 |  |  |  |
|  | 186 | chr8:19,613,104-19,613,262 |  |  |  |
|  | 187 | chr8:22,781,766-22,781,898 |  |  |  |
|  | 188 | chr8:26,981,949-26,982,054 |  |  |  |
|  | 189 | chr8:29,920,426-29,920,585 |  |  |  |
|  | 190 | chr8:5,174,932-5,175,084 |  |  |  |
|  | 191 | chr8:8,082,357-8,082,456 |  |  |  |
|  | 192 | chr9:10,046,566-10,046,745 |  |  |  |
|  | 193 | chr9:15,558,819-15,559,001 |  |  |  |
|  | 194 | chr9:19,473,220-19,473,396 |  |  |  |
|  | 195 | chr9:21,544,701-21,544,877 |  |  |  |
|  | 196 | chr9:24,434,437-24,434,575 |  |  |  |
|  | 197 | chr9:5,790,909-5,791,033 |  |  |  |
|  | 198 | chrZ:26,259,592-26,259,752 |  |  |  |
|  | 199 | chrZ:36,340,716-36,340,889 |  |  |  |
|  | 200 | chrZ:53,370,028-53,370,177 |  |  |  |
|  |  |  |  |  |  |
| * repeated sequences, redundancies and unknown or random chromosome locations are excluded from the list | | | | | |
