# Supplementary material for: The Early Apoptotic DNA Fragmentation Targets a Small Number of Specific Open Chromatin Regions
Source: PLoS One. 2009 Apr 6;4(4):e5010. doi: 10.1371/journal.pone.0005010 (PMC2661134; doi:10.1371/journal.pone.0005010)
Supplement: Table S2 — (0.04 MB DOC) [file pone.0005010.s005.doc]

**Table S2**

Analysis of the frequency of the terminal nucleotides of the cloned fragments from MNase degradation.

cut

cut

|  | | | | | | |  | | | | | | |
| --- | --- | --- | --- | --- | --- | --- | --- | --- | --- | --- | --- | --- | --- |
| **5'-end** |  | I | II | III | IV |  |  | IV | III | II | I | **3’ -end** | |
|  | **A** | 52 | 25 | 25 | 46 |  | **A** | 45 | 29 | 8 | 98 |  |  |
|  | **C** | 6 | 25 | 91 | 63 |  | **C** | 21 | 33 | 108 | 13 |  |  |
|  | **G** | 7 | 119 | 37 | 18 |  | **G** | 49 | 90 | 25 | 11 |  |  |
|  | **T** | 106 | 8 | 25 | 48 |  | **T** | 54 | 17 | 29 | 47 |  |  |
|  |  |  |  |  |  |  |  |  |  |  |  |  |  |
|  |  |  |  |  |  |  |  |  |  |  |  |  |  |

The MNase fragment orientation is 5’-> 3’. The results obtained at the ends of the fragments cloned were separated. In the 1st position there was found a striking preference for T and A, in the 2^nd^ and 3^rd^ position for G or C.
