# Supplementary material for: The Early Apoptotic DNA Fragmentation Targets a Small Number of Specific Open Chromatin Regions
Source: PLoS One. 2009 Apr 6;4(4):e5010. doi: 10.1371/journal.pone.0005010 (PMC2661134; doi:10.1371/journal.pone.0005010)
Supplement: Table S3 — (0.04 MB DOC) [file pone.0005010.s006.doc]

**Table S3**

Origin and description of the genes reported in Figure 5^a^.

|  |  | |  | |  |
| --- | --- | --- | --- | --- | --- |
| ***Gene*** | | ***origin*** | | ***description*** | |
| *HOXD8* | *Homo sapiens* | | homeobox D8 | |  |
| *Filip1* | *Homo sapiens* | | filamin A interacting protein 1 | |  |
| *COL14A1* | *Gallus gallus* | | collagen type XIV alpha 1 (undulin) | |  |
| *GRB14* | *Homo sapiens* | | growth factor receptor-bound protein 14 | |  |
| *PLA2G4B* | *Homo sapiens* | | phospholipase A2 group IVB | |  |
| *Tmem87a* | *Mus musculus* | | transmembrane protein 87A isoform 2 | |  |
| *NEUROD1* | *Gallus gallus* | | neurogenic differentiation 1 | |  |
| *CERKL* | *Homo sapiens* | | ceramide kinase-like isoform a | |  |
| *ITGA4* | *Homo sapiens* | | integrin alpha 4 precursor | |  |
| *TCERG1L* | *Homo sapiens* | | transcription elongation regulator 1-like | |  |
| *C13orf15* | *Homo sapiens* | | response gene to complement 32 | |  |
|  |  | |  | |  |

^a^ The identification of most genes were based on the orthologous human

(in one case mouse) genes.
